# *Amblyomma mixtum* free-living stages: Inferences on dry and wet seasons use, preference, and niche width in an agroecosystem (Yopal, Casanare, Colombia)

**DOI:** 10.1371/journal.pone.0245109

**Published:** 2022-04-06

**Authors:** Elkin Forero-Becerra, Alberto Acosta, Efraín Benavides, Heidy-C. Martínez-Díaz, Marylin Hidalgo

**Affiliations:** 1 Faculty of Sciences, Departament of Microbiology, Pontificia Universidad Javeriana, Bogotá, D.C., Colombia; 2 Faculty of Sciences, Departament de Biology, UNESIS (Unidad de Ecología y Sistemática), Pontificia Universidad Javeriana, Bogotá, D.C., Colombia; 3 Facultad de Ciencias Agropecuarias, Grupo de Investigación Epidemiología y Salud Pública, Universidad de La Salle, Bogotá, Colombia; 4 Faculty of Sciences, Laboratorio de Bacteriología Especial, Departament of Microbiology, Pontificia Universidad Javeriana, Bogotá, D.C., Colombia; Beni Suef University Faculty of Veterinary Medicine, EGYPT

## Abstract

The formulation of effective control strategies for any pest species generally involves the study of habitat use and preference and niche width in anthropogenically transformed natural landscapes. We evaluated whether the use, habitat preference, and niche range of the *Amblyomma mixtum* tick changed between stages, habitats, and seasonality (dry-wet seasons 2019) on a farm in Yopal (Casanare, Colombia). The presence and relative abundance of free-living larvae, nymphs, and adults was quantified in four different habitats according to the type of vegetation cover (Riparian Forest, Cocoa Crop, King Grass Crop, and Star Grass Paddock). Habitat availability was estimated, environmental variables were analyzed, and various indices of habitat use and preference, and niche width were calculated. *A*. *mixtum*’s habitat use and preference, and niche width changed between stages, habitat types, and time of the year. The total abundance of *A*. *mixtum* was an order of magnitude greater in the dry season than the wet season. In the dry season, all stages used all habitats, while *A*. *mixtum* adults used all the habitats in both seasons. In the dry season, nymphs and larvae preferred three out of the four habitats, while adults preferred the King Grass Crop. In the wet season, nymphs and larvae preferred two habitats, whereas the adults preferred the King Grass Crop. The value of the niche width index was high for larvae, nymphs, and adults in the dry season, while it was high only for adults in the wet season. Thus, *A*. *mixtum’s* vast environmental tolerance and niche breadth allows the species to use and colonize changing habitats (unstable or temporary) with fluctuating environmental conditions (e.g., King Grass Crop), potentially keeping a stable population over time and making it an extremely resistant species. However, the wet flooding season in Yopal may exceed *A*. *mixtum*’s stages’ tolerances.

## Introduction

Animals’ habitat use, preference, niche width, and resource selection allow researchers to explain a given organism/species abundance, spatial distribution, and evolutionary processes to infer its ecological requirements (animal–habitat relationships) in a changing world. Including the effects of human activities on wildlife’s ability to colonize and use specific areas [1–3].

Habitat is defined as the site that meets all the conditions and resources necessary for survival, development, reproduction, and the establishment of local populations [4]. Habitat is also the spatially limited area where abundance or other population parameters (rate of growth = R) differ from those of other localities or contiguous patches [5]. For example, in host-parasite relationships, the host is both the site and habitat that offers the resource (e.g., blood source to a tick species) where different host types will define the tick’s population abundance, not implying that all the available hosts in the habitat will be used. Resources have been described [6] as units with particular attributes or environmental variables within the vegetation cover types encountered, used, or preferred by an animal. It will instinctively determine which resource it will use based on cost-benefit. Thus, a utilized resource unit implies the animal’s investment, whether time or energy expending, traveled distance, or duration in the unit/habit [3].

A resource can be available or unavailable in an organism’s habitat. An animal can potentially encounter an available resource unit (spatially limited by the researcher) [3], depending on the presence or absence of physical and biological limiting factors in time and space, that could prevent its establishment, survival, development, or reproduction [2]. Availability is defined by the proportional occurrence of a resource’s discrete levels (e.g., an area covered by different vegetation types or habitats). Comparing used to unused resource units to available resource units allows inferring on selection and preference [3]. Thus, habitat preference is understood as a consequence of habitat selection by a population’s subset or a population’s individual stage’s non-random asymmetric use of a resource over all possible resources found in other available habitats [5], depending on the organism’s requirements. Therefore, habitat preference must be inferred at the population level (many individuals choosing the same physical or biological resource in a habitat over the availability of others) [2].

Essentially, an animal’s habitat preference is assessed by its relative abundance in the habitats it uses and their resources according to the availability of comparable habitats it can encounter. The diversity of resources used and conditions needed in a habitat by any organism or group of organisms to survive and reproduce is its niche width [6] (amplitude or breadth). Hence, species abundance and spatial distribution are attributed to its niche amplitude (as a proxy) [6]. In any habitat, each factor can have a lower or upper limit, anything below or above these limits will hinder the species from growing or cause its elimination (exceeding its tolerance limits). These limits are the optimum range for species performance, survival, and positive population growth rate. A factor’s range between minimum and maximum values represents the specie’s ecological amplitude for that factor [7]. Species with a vast niche breadth are highly adaptable; they can use a broader spectrum of resources, even with limited efficiency, according to scarcity or availability of those resources in the habitat, easily adjusting to changes in the environment. These generalist species (e.g. some *Amblyomma* tick species) could potentially live in a broad range of temperature or humidity conditions, attaining higher population abundance and occupying several different habitats (resource possibilities) [6]. Although describing and determining any specie’s ecological niche (infinite niche dimensions) is challenging, a few significant variables and the species response to them (survival, abundance) may be sufficient to measure its established niche and infer its niche width [6]. Depending on the species’ niche, individuals actively seek out and select habitats that provide an adequate range of conditions and resources, essential to the population’s preservation and persistence [8]. However, natural habitats are constantly changing, and so are the species. Human activities, for instance, benefit synanthropic species’ spatial distribution and abundance [9] by expanding their established niche [10], particularly in agroecosystems [11]. The favored species (e.g., some tick species) increase their dispersion capacity to colonize different habitats consequently, decreasing their extinction probability [12]. Forest degradation and fragmentation, for example, have prompted the occurrence of *Amblyomma aureolatum* in urban areas of the city of São Paulo (Brazil) [13,14].

Ticks of the *Amblyomma* genus have been recognized as vectors of rickettsial agents to humans and animals in a number of countries; thus, the importance of their study [15]. Some examples of research on ixodids of vector-pathogen-host relationships are *Ixodes scapularis* [16]; *Amblyomma fuscum* [17]; *Amblyomma americanum* [18]; *A*. *aureolatum* and *Amblyomma ovale* [19]; *Amblyomma triste* [20]; *Ixodes ricinus* [21]; *Ixodes pacificus* [22]; *Amblyomma tuberculatum* [23]; *Rhipicephalus microplus* [24], *Amblyomma maculatum* [25], and *Amblyomma sculptum* [26–28] and *Amblyomma tonelliae* [29], both from the *Amblyomma cajennense* species complex [30]. In Colombia, *Rickettsia rickettsia* infection has been reported in *Amblyomma patinoi* (Villeta, Cundinamarca [31]), *A*. *cajennense* sensu lato (La Sierra and Rosas municipalities, Cauca, [32]), and *Amblyomma mixtum* in several regions, including Yopal (Casanare [33]). *A*. *mixtum* has been reported parasitizing humans [34,35].

There is a trend towards studying the tick’s eco-epidemiology, particularly the limits of their geographic distribution, using mathematical modeling of their niche based on records of local presence and seasonal abundance [36]. However, the absence of observations cannot be interpreted as the specie’s non-existence in the site [37].

Worldwide, 137 species of *Amblyomma* have recognized, most of them are established in the lands of Gondwanian origin and the highest number of species occurs in the Neotropical region [38]. In particular, *A*. *cajennese* was previously considered as a species with a unique evolutionary origin and its geographical distribution ranged from Northern Argentina to South Texas [39]. Recently, [30,40] established *A*. *cajennese* as a species complex because of allopatric speciation with up to six recognized species: *A*. *cajennense* sensu stricto, *A*. *sculptum*, *A*. *tonelliae*, *Amblyomma interandinum*, *A*. *patinoi*, and *A*. *mixtum*. In Colombia, the current known distribution of *A*. *patinoi* is Villeta municipality (Cundinarmarca) [30], while *A*. *mixtum* has been reported the Orinoquia region and Caldas [41]. However, there are no studies in Colombia that clearly indicate *A*. *mixtum*’s limit of altitudinal distribution in the Eastern Cordillera or explanations based on empirical data of its absence in the Bogota savannah.

Several field studies have evaluated the *Amblyomma* ticks’ seasonal population dynamics and abundance (see [27,42–47]). However, few have approached the use of resources in the habitat or the inference of their niche range on a local scale. Although some factors that could affect *A*. *mixtum*’s habitat use have been studied at the local [48] or laboratory level [49], or modeled on a regional [50] or continental scale [51], there is still a lack of empirical information (tolerance to extreme abiotic variables values, and its interaction with biotic factors) that evaluates and integrates aspects of this tick’s use, habitat preference, and niche range in an agricultural system and whether the non-parasitic stages and population respond differently over time (seasonality). Detailed empirical data is needed to add biological realism levels to understand that not all ticks and their hosts behave the same way in all environments [52]. According to a recent epidemiological study in the Matepantano farm in Yopal, Casanare (Hidalgo, et al., unpublished information), there is a high proportion of the human population (92%, 77/84) with antibody titers to *Rickettsia* spp. due to their exposure to ticks on the farm. Considering the scarce information existing between non-parasitic *A*. *mixtum*’s stages and their relationship with resources and habitat conditions, we were prompted to investigate the *A*. *mixtum* tick population’s use of and preference for four different habitats and their niche width within the farm. We compared the dry season in February 2019 and the wet season in August 2019 to determine if the seasonal variation (precipitation, humidity, and temperature) altered *A*. *mixtum* use and preference of the habitats and the niche width (intraspecific variation), as suggested for other animal species [3].

## Materials and methods

### Habitat selection and sampling details

**Fig 1:**
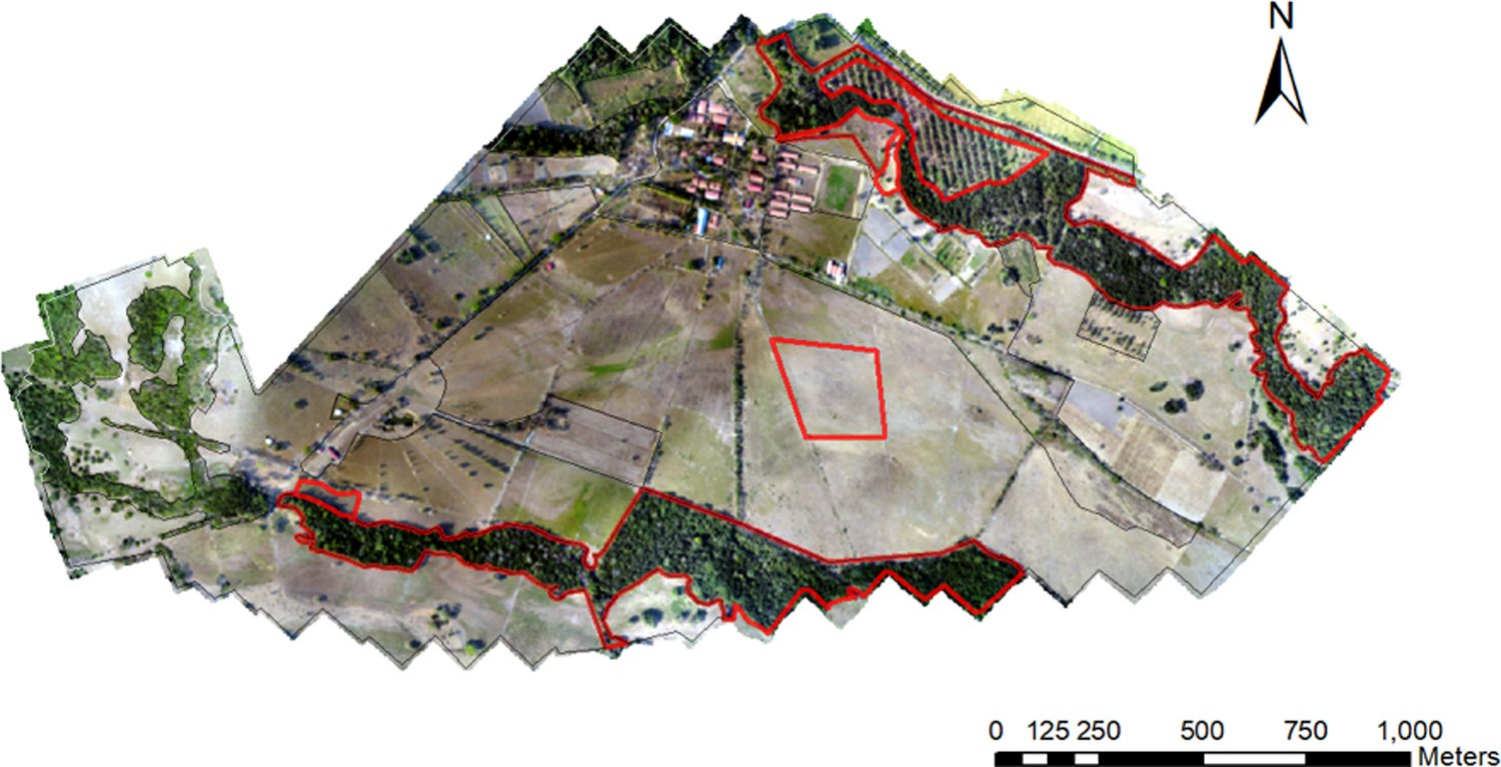
Aerial view of the Matepantano farm study area from the orthomosaic taken by a dron imaging service. Red lines encircle areas of the potential habitats to select the sample sites.

The work was carried out within the denominated Matepantano farm, an area surrounding part of the Hacienda Matepantano’s main installations, a private-owned land where academic agricultural spaces and activities occur (Vereda Matepantano, Yopal, Casanare, Colombia). The studied biome is known as a tropical rainforest (a landscape characterized by gallery forest fragments around rivers and creeks with intersparsed savanna of different sizes) in the Colombian Piedemonte Llanero (lowland foothills), a kind of the Andean forests. Non-protected species were sampled. The field collections were possible thanks to the permission of the Colombian Ministry of the Environment (001–2018). The farm is located at an altitude between 256–270 m.a.s.l., a latitude between 5.32482 and 5.31917 N, and longitude between -72.29163 and -72.28691 W (detailed locations are presented in the “Generalities of the Compared Habitats” section). It is delimited by gallery forest corridors, which are home to wildlife. Initially, a drone imaging service was hired (Fig 1) to take hundreds of successive aerial images with a digital camera mounted on a Phantom 4 Advanced DJI drone from an altitude of 150 m (S1 Appendix). The pictures were used to create an orthomosaic of the study area (domain) in ArcGIS Desktop^®^ (Esri, Redlands, CA, USA).

**Fig 2:**
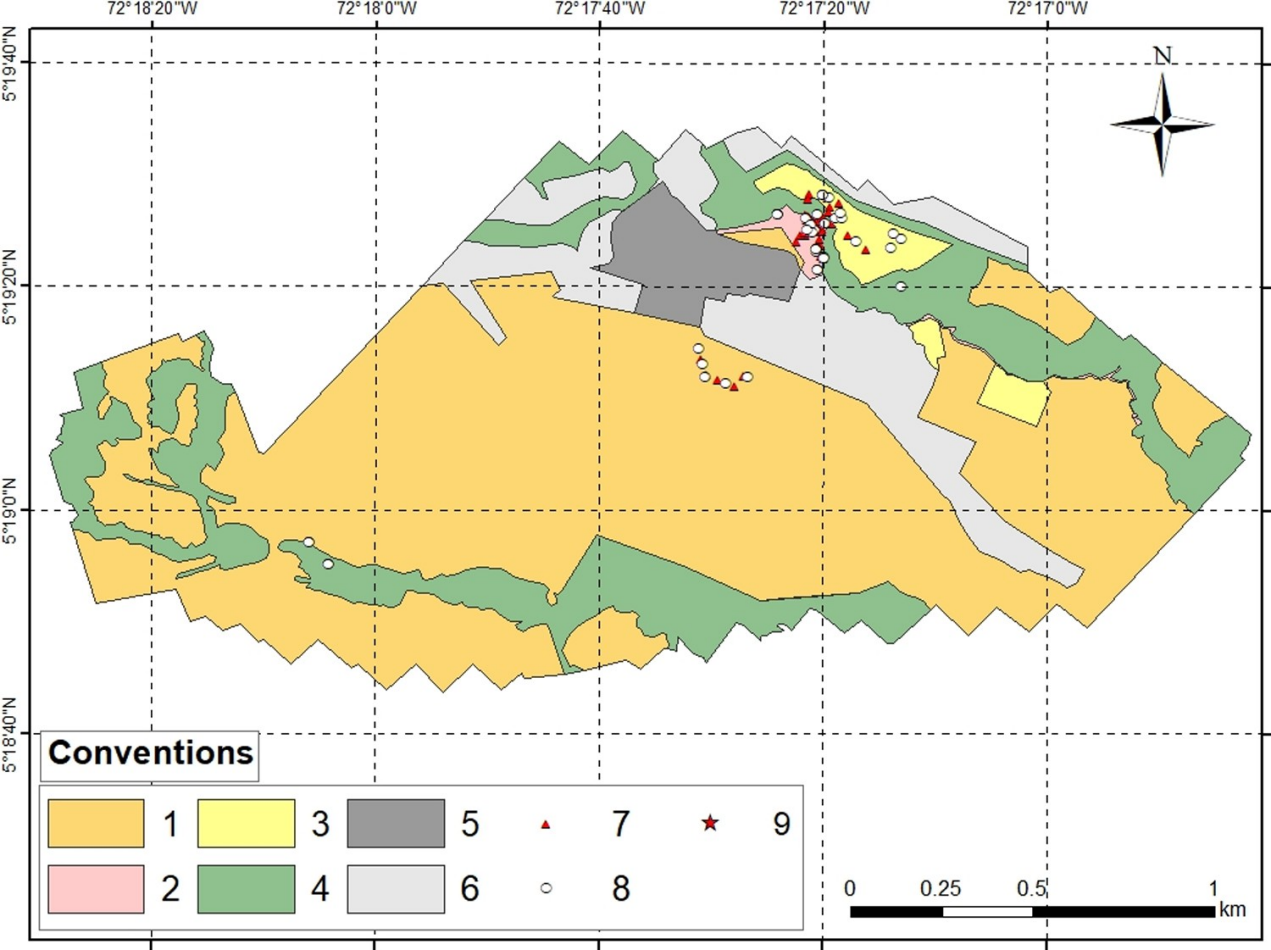
Derived map of the Matepantano farm study area and the four selected habitats. The study area is about 3.2 km^2^ representing a 6% of the total area of Matepantano estate. Conventions: 1- Star Grass paddocks (1’955,090 m^2^); 2- King Grass crop (22,416 m^2^); 3- Cocoa crop (81,341 m^2^); 4- Riparian Forest (646,099 m^2^); 5- Main buildings area (112,833 m^2^); 6- Other area (386,007 m^2^); 7- CO_2_ traps; 8- Dragging by cloth transects; 9- Regional localization of the Matepantano farm. Note: All CO_2_ traps and dragging by cloths transects with GPS data for both the dry and wet seasons are represented.

The spatial scale was determined to allow us to capture the domain’s maximum spatial heterogeneity (*discrete vegetation types*) produced by anthropic intervention processes, like livestock, crops, and pastures, to see their effect on *A*. *mixtum*’s use of resources. The different habitats were defined and delimited on the orthomosaic according to the type of vegetation and the different patches of vegetation within each habitat (Fig 2). Then, ArcGIS was used to calculate the Matepantano farm’s total area (3’203,786.9 m^2^) and the area of each of the four selected habitats. Only those areas (discrete patches or paddocks) of habitat where tick sampling was conducted were considered (previously confirmed presence of *A*. *mixtum* in pre-samples).

The areas finally selected were the following: riparian forest, 450,552 m^2^ of a total of 646,099 m^2^; cocoa crop (*Theobroma cacao)*, 59,940 m^2^ of a total of 81,341.3 m^2^; Star Grass paddock, 54,968 m^2^ of a total of 1’955,090.4 m^2^; and King Grass crop, 13,245 m^2^ of a total of 22,416.3 m^2^. These four areas, called “available area” in this study, were areas with potential to be used by the *A*. *mixtum* tick population. After performing a terrain survey and obtaining the drone-generated orthomosaic image, we selected four main agroecosystems habitats in the Matepantano farm based on the following: 1) that EB (coauthor) has been studying *A*. *mixtum* ticks and other ectoparasites in the Matepantano farm for the past six years, recording only the presence of *A*. *mixtum* adults in all four agroecosystems habitats; and 2) that ranchers and locals working at the Matepantano farm have seen some wild and ocationally domestic animals moving between these agroecosystems habitats. This “available area” is essential in determining the observed use in each habitat (presence and abundance of ticks) and defining habitat preference and niche width. All four habitats were equally represented in the habitat availability area, except for the Star Grass paddock, which we did not want to over-represent in the habitat availability, given its higher proportion in the domain area (Fig 2).

The mathematical calculations (indices) used the same values for the dry and wet seasons in the four available areas, given that the same sites were sampled in time. Some areas within the Matepantano farm were not sampled, including buildings for academic, recreational, eating, and lodging activities and some of the four habitats’ discrete patches (386,007 m^2^) (Fig 2). Within each habitat, a sampling of free-living ticks was performed, from February 8 to 12, 2019 during the dry season (November-March), and from August 16 to 19, 2019 during the wet season (April-October). Seasonality alters the relationship between a species and the use of the resource (habitat) [3]. The CO_2_ trap and white flannel dragging transect sampling units (grouping all the tick individuals captured by one trap or one transect) were located at convenience within each habitat category, following biological criteria (tick biology and activity pattern and the probabilities of finding a human carrier), with replicates varying for each habitat from four to 12, according to area size and shrub density. The habitat fragments, patches, and paddocks, chosen and sampled from the photomosaic, favored those within a radius of approximately 500 m from the Matepantano farm’s buildings, including the places frequently used for agropastoral practices (Figs 1 and 2). The number of traps and transects were fairly similar between habitats related to the sampled area to facilitate comparison of relative abundances. Random sampling was not used for the location of the sampling units (trap or transect) in the vegetation types because this usually results in few or no observations of habitat use [3].

### Generalities of the compared habitats

#### Riparian forest

(5.32224 N and 72.28696 W). This habitat was a gallery forest bordered by a permanent creek and dominated by *Attalea butyracea* (Arecaceae) palm adults and juveniles, a closed canopy, and an open understory (Table 1). There was no apparent change in vegetation structure between summer and winter (stable structure), with saturated soil in winter and litter in both seasons. Tick abundance sampling was conducted between 2 and 8 m into the forest, outside the edge zone.

**Table 1:** Structural characteristics of the four habitats-selected where ticks were collected.

	Riparian forest	Cocoa crop	King Grass crop	Star Grass paddocks
**Dominant terrestrial vegetation**	Palm (*Attalea butyracea*). Low litter with presence of nuts, tree branches, litter debris, and fungi.	Cocoa, acacia, banana trees. Acacia leaves dominate the litter with fruits and tree branches.	King grass dominates this area with some scattered trees and thorny bushes. No litter.	Star grass (*Cynodon* sp.) and kermes oak. Litter was scarce with predominance of grass dry leaves. Soil is floodable by rains, but humidity occured during a 24 h period.
**Natural light availability (%)**	25	50	100	100
**Number of vegetation strata**	4	3	1–2	2
**Litter depth (cm)**	2.9 (±1.6)	6.8 (±3.5)	8.5 (±2.2)	2.8 (±2.8)
**Litter humidity (%) in the dry season**	56.4 (±6.5)	47.4 (±5.1)	62.9 (±8.2)	39.9 (±6.7)
**Soil dominant fraction**	Clay soil with some sand.	Clay soil (alfisol type). There are rocks at deeper digging. The soil has acidic pH.	Clay soil with few silt and fine sand soils.	Clay soil.

#### Cocoa crop

(5.32312 N and 72.28691 W). This habitat was a *Theobroma cacao* crop. The farm used the *tresbolillo* technique to plant the cocoa. It entails placing the plants in a triangular shape with a distance of 3.5 x 3.5 m between them. The crop was mixed with *Acacia* trees to provide shade (Table 1) and some banana plants. In the studied dry season, the crop soil was dry (cracked), with leaf litter and dehydrated plants produced by a couple of months of drought (December-January). In the studied wet season, the soil was saturated, and the vegetation was green. Leaf litter was observed in both dry and wet seasons.

#### King grass crop

(5.32482 N and 72.28704 W). This habitat was a *Pennisetum hybridum* King Grass Crop. In the studied dry season, the grass was dry (yellowish-orange appearance), sometimes broken, but dense, with a height between 1 and 2 m and interspersed with thorny shrubs and very few scattered trees (Table 1). In the studied wet season, the same pasture was green (1.8 m high), and the soil was wet from rainfall. The search for ticks was conditioned by accessibility and the possibility of placing the bases of the CO_2_ traps in the dense grassland. However, the collection points were distributed broadly over the habitat patches.

#### Star grass paddock

(5.31917 N y 72.29163 W). This habitat was a with *Cynodon nlemfuensis* African Star Grass paddock devoted to cattle breeding (*Bos taurus indicus*). The grass in the paddocks reached a height between 20–40 cm and had some scattered bushes (Table 1). In the studied dry season, the paddock’s grass was dry (yellowish appearance). The soil was dry and cracked due to drought. In the studied rainy season, the grass was green, and the soil partially flooded a few centimeters (<10 cm after rain); however, it percolated quickly from one day to the next. The CO_2_ traps were placed in flooded and non-flooded areas in this habitat only. However, distinction was not made for the total number of ticks collected.

The four habitats’ (Riparian Forest, Cocoa Crop, Star Grass Paddock, and King Grass Crop) vegetation’s structural characteristics (Table 1), including dominant species, light availability, number of strata, depth of leaf litter above the soil, percentage of leaf litter moisture, and dominant soil fraction, were qualified and quantified (details in S2 Appendix) in the sites where ticks were sampled.

### Collection of *A*. *mixtum* specimens in the environment

To define habitat use, habitat preference and relative niche width by *A*. *mixtum* free-living stages and the relative abundance response variable of individuals in each habitat (presence indicating resource use and tolerance to abiotic conditions in the habitat) were used as a proxy for resource selection in the habitat. Based on previous field experiences, it was decided to capture free-living stages of *A*. *mixtum* through two techniques, CO_2_ traps (dry ice) and white flannel dragging transects (see [53–55]).

#### Use of CO_2_ traps

The details of these traps’ construction before their placement are presented in S3 Appendix. According to vegetation cover, the traps were placed in relatively flat sites and areas, which according to locals were frequented by domestic and wild animals, and considering their proximity to transited areas (corridors) or roads used by campus workers and students. The number of CO_2_ traps placed in each habitat in the dry (February) and wet (August) seasons ranged between 4–8 and 5–12, respectively. CO_2_ traps were used once per habitat type in each season, separated by a minimum distance of 5 m. Sampling was conducted between 8:00 am and 1:30 pm, and 2:30 and 4:30 pm, or until the dry ice was consumed. The traps were checked every 30 minutes. The ticks caught on the double-faced tape and those nearby, were collected. Twenty-four traps were placed in the dry season and 31 in the wet season (a higher number was needed in the wet season because of the shrub density in some areas) (S1 and S3 Tables). The captured specimens were collected with entomological tweezers, deposited in 50 mL centrifuge tubes with 70% ethanol, and labeled. Later, in the Veterinary Parasitology Laboratory at the University of La Salle (Bogotá, D.C., Colombia), the specimens were identified and counted according to genus or species, sex, and stage. Subsequently, they were stored in 1,5 mL microcentrifuge tubes with 70% ethanol in a freezer at -80°C at the Special Bacteriology Laboratory of the Pontificia Universidad Javeriana (Bogotá, D.C., Colombia).

#### White flannel dragging transects

Details of this activity’s outputs and the size of the quantified transects are presented in the S3 Appendix. The sampling was done between 8:00 am and 1:30 pm, or between 2:30 and 4:30 pm, covering several habitats in one day, some in the morning and others in the afternoon. The transects were separated from each other by at least 5 m and approximately between 10 to 50 m from a CO_2_ trap. A transect never took place in the same path in the same season. All captured ticks by a white flannel were removed with entomological tweezers and deposited in 50 mL centrifuge tubes with 70% ethanol before starting a new transect. The white flannel was dragged for about 5–10 min (a time equivalent to approximately 60–90 m of lineal sampling of one transect), making stops roughly every 20 m to collect any tick attached to the cloth. A few minutes to over half an hour was needed to remove all the captured ticks in a transect. A minimum number of four transects per habitat was performed, once in each season of the year. In the riparian forest habitat, flannel trails were performed when possible, following the paths created by the wildlife and tree base or herbaceous creeping vegetation (<70 cm). The total number of flannel transects was 22 for each time of year, for a total of 44 in the two seasons (S2 and S4 Tables). Two teams of three researchers simultaneously carried out the sampling, using the two capture techniques (CO_2_ traps and transects) in each habitat. Each team initially installed the corresponding CO_2_ traps in the four habitats. Flannel dragging was carried out in the defined transects between the time intervals of the trap inspections.

### Taxonomic identification of specimens

Stereoscopy was used to identify the morphological characteristics in adults and some characteristics in nymphs. Light microscopy was used to identify certain morphological characteristics in larvae by mounting up to five specimens between one lamina and one lamella using Hoyer’s solution (a subsample of the specimens captured in each habitat and season). The taxonomic keys of several authors were used only for the genus *Amblyomma* in the adult [56–60], nymph [61,62] and larval [63] stages. The descriptions made by [30] were used to define the *A*. *mixtum* species only in adult specimens.

Molecular identification of selected *A*. *mixtum* stage samples were based on two mitochondrial genes (large subunit ribosomal RNA gene or 16S rRNA gene, and Cytochrome C Oxidase subunit 1 gene or COI gene) and one nuclear gene (Internal Transcribed Spacer 2 gene or ITS-2 gene). For this purpose, we selected for DNA extraction seven female individual specimens, six male individual specimens, two male pools of up to two individual specimens each, three larva pools (L1, L2, and L3) of up to 34 individual specimens each, and two nymph pools (N1 and N2) of six individual specimens each from the wet season (August/2019) and one nymph pool (N3) of six individual specimens from the dry season (February/2019). In addition, four larvae pools (P44, P82, P140, and P154) of 30 individual specimens each plus one larvae pool (P77) with only nine individual specimens and two nymph pools (P47 and P145) of 15 individual specimens each from the dry season (February/2019; see S4 Appendix) were selected for DNA extraction. In total, 28 samples of all tick stages and both dry and wet seasons were processed. DNA was extracted using a DNeasy Blood & Tissue Kit 69506 (250) (Qiagen, Hilden, Germany) modified with an overnight digestion in DNAzol at 56°C and stored at 4°C [87] until being used in PCR assays.

A ≈460 bp fragment of the large subunit ribosomal RNA (16S-rRNA) gene with the primers 16S-F (5′-CCGGTCTGAACTCAGATCAAGT-3′) and 16S-R (5′GCTCAATGATTTTTTAAATTGCTGT-3’) was targeted using a published PCR protocol [88]. Also, a ≈700 bp fragment of the Cytochrome Oxidase subunit I (COI) using the primers LCO1490-(F) (5′-GGTCAACAAATCATAAAGATATTGG-3′) and HCO2198-(R) (5′-TAAACTTCAGGGTGACCAAAAAATCA-3′) was carried out according to [89]. Finally, a ≈1,000 bp fragment of the ribosomal Internal Transcribed Spacer 2 (ITS-2) region was amplified by cPCR using the primers ITS2-(F) (5′-CCATCGATGTGAAYTGCAGGACA-3′) [90] and MCLN-(R) (5′-GTGAATTCTATGCTTAAATTCAGGGGGT-3′) [91] following the next PCR conditions: activation at 94°C x 3 min followed by 35 cycles of denaturation (94°C x 30 s), annealing (52.6°C x 30 s), and extensión (72°C x 1 min), with a final elongation step at 72°C x 5 min. All PCR assays used the same Taq polymerase (GoTaq® Green Master Mix, M7122, Promega, Madison, WI, USA).

Amplified PCR products were visualized in an UV transilluminator (Molecular Imager^®^ Gel Doc XR System Modular, Bio-Rad, Hercules, CA, USA) after running an agarose gel (2%) (SeaKem® LE Agarose, Lonza by Thermo Fisher Scientific, Waltham, MA, USA) horizontal electrophoresis (Bio-Rad Wide Mini-Sub Cell GT Systems and Bio-Rad PowerPac 200, Hercules, CA, USA) containing 0.003% staining solution (SYBR^TM^ Safe DNA gel stain, Invitrogen by Thermo Fisher Scientific, Waltham, MA, USA) in TBE 1X at 100 V for 45 minutes. Subsequently, amplicons from each target gene for one female (V7, King grass crop, August/2019), one male pool (V4, King grass crop, August/2019), one larvae pool (L3, King grass crop, August/2019) plus up to five larva pool (P44, Riparian forest; P77, P82, P154, King grass crop; P140, Cocoa crop; February/2019) and one nymph pool (N3, King grass crop and Cocoa crop, February/2019) plus two nymphs pools (P47, Riparian forest; P145, Cocoa crop; February/2019) were randomly selected for bidirectional Sanger sequencing at the Gencore Laboratory (Universidad de los Andes, Bogotá, D.C., Colombia).

The obtained DNA sequences were assembled using BioEdit v7.0.5.3 (http://www.mbio.ncsu.edu/BioEdit) and then aligned through the nucleotide BLASTn program provided by the National Center for Biotechnology Information (NCBI) using the Clustal algorithm [92] for comparison with deposited reference sequences in the GenBank (https://blast.ncbi.nlm.nih.gov). A phylogenetic analysis was performed using the Maximun Livelihood method based on Tamamura-3 parameter model for 16S rDNA and ITS-2 gene sequences and the Hasegawa-Kishino-Yano model for the COI gene sequences and 1,000 bootstrap replicates, as well as their comparison with available *A*. *mixtum* gene sequences in the GenBank.

### Inference of use, habitat preference, and niche width

Based on the resource selection by animals theory [3], this study assumed that the presence of ticks in a habitat might be possible because: 1) ticks may use one or more resources (even if we do not know exactly which or what those resources are, given the lack of biological data on the species), and 2) ticks can tolerate the habitat’s environmental conditions where we found them. Therefore, we used a paired used/availability habitat design with four discrete levels of habitats with different types of vegetation: tropical, riparian evergreen forest, tall grass plantation (King Grass), grazing grass (Star Grass pastures), and cocoa plantation.

We quantified the proportional availability area of a particular habitat. Then, we quantified the observations of use (abundance) in that habitat at the population level. We performed a graphical analysis with these two variables, comparing the four discrete vegetation types’ proportional use against each habitat’s proportional availability [64]. Using the HaviStat 2.4 free-access program [65,66], we explored the visual data to infer relative use and habitat preference between the categories compared (vegetation types, months of different seasons). The Havistat program has been successfully employed in biological research [67–70]. The greater the disproportion between these two variables in the graph, the higher the preference [69]. Ultimately, a preference was determined when the abundance exceeded the potential habitat area to be used (availability); otherwise, there is use and no preference [2,69]. Resource selection can be established when the resource use is disproportionately greater than resource availability [71]; this is assumed correlated with the animal’s fitness [72].

To confirm the graphic approach to habitat use (presence of the species or its stages), habitat preference (asymmetric distribution of resources), or non-use (non-presence of the species in the habitat), five preference indices were calculated using the HaviStat 2.4 program, including Duncan’s index [73], Ivlev’s Electivility Index [74], Bailey confidence interval [75], Alpha index [76,77], constant resource rate (*Constant Resources*) [78], and Interpretation of II ([79]). The HaviStat 2.4 program allows to: 1) check if the sample size is adequate (power, significance level, G-test, Chi-test, and Cherry-test [80]), which is useful for generating inferences at the population level and proposing management strategies [3]; 2) statistically compare field data (95% confidence intervals); and 3) estimate five niche amplitude indices (Standardized Shannon [81], Ivlev’s Electivility Index [74], Levins index [82], Standardized Levins Index [83], and Levins Index Standardized modified by [84]).

The niche width indices were applied to infer whether the habitat resources were used or exploited uniformly (or asymmetrically) by the observed species and the conditions that allow its survival there. In this study, we measured how much wider the spectrum of resources used was in the habitat and how a species could potentially live in a broad range of conditions; this was indicated by its relative abundance (proxy), and the number of habitats inhabited [85]. Regarding the latter, resources and conditions should be considered as a whole, without specific differentiation, which is impossible.

By reaching a consensus among the indices, it is possible to differentiate whether a species uses a habitat (resources) or whether it strongly prefers or avoids another. Resources are avoided in a habitat when resource use (relative abundance) is disproportionately less than expected, based on the availability of that resource (potentially available habitat) [71]. Consequently, animals may still use even avoided resources.

### Mammal species seen by locals in the four habitats

First, the literature review was used to identify the mammal species most likely to be found in the study area. Posters were made with photographs of these mammal species. They were shown to the people who work, live, or manage the Matepantano farm to identify them photographically (n = 75 people). *In situ* photographic evidence and videos taken by locals were also used to confirm the species. From all the collected data, a list was made of the domestic and wild hosts potentially present on the farm (see “Potential hosts seen by locals” in the Results section). It was contrasted against the mammal species that, according to the literature, are commonly parasitized by *A*. *cajennense* s.l. Similarly, qualitative indications were obtained of the relative abundances of host populations for both dry and wet seasons. This information was collected for each of the four habitats compared. Hosts that usually approach the campus buildings and residences were also included.

### Climatological analysis of the region

The maximum and minimum range of oscillation of different key environmental variables was calculated for *A*. *mixtum*, representing its ecological amplitude (niche width). To this end, a 29-year analysis was made of the climatological data available (1975–2004) from two Colombian Institute of Hydrology, Meteorology, and Environmental Studies (IDEAM, acronym in Spanish) meteorological stations within a 20 km radius from the Matepantano farm: the main automatic telemetry weather station at the Yopal airport (code 35215020), and the Yopal conventional pluviometric station (code 35210020, 03.05 N, 76.33 W, 1,205 m.a.s.l.). The enquiry was made in 2019. This information was complemented with a multi-year analysis (1981–2010) provided by the IDEAM (available at http://atlas.ideam.gov.co/visorAtlasClimatologico.html). Because of its spatial scale, we termed this climate analysis “regional level.”

#### Local weather and microclimate information

A permanent station for continuous climate recording is located in the cocoa crop habitat at the Matepantano farm. This station, called Davis, has registered temperature, relative humidity, solar radiation, solar energy, rainfall, and wind speed for seven years (2012 to 2019 every 30 minutes) using a Davis-Vantage Pro2 unit located at an altitude of 1.2 m and approximately 300 m from the Matepantano farm’s main buildings. We also calculated the range of oscillation (maximum and minimum value) of different key environmental variables for the tick (ecological amplitude of *A*. *mixtum*). Because of its spatial scale, we termed this climate analysis “local level.”

To determine the range of daily oscillation experienced by ticks at the microhabitat level, the temperature, humidity, and solar irradiance were measured *in situ* in the Cocoa Crop, Star Grass Paddock, and Riparian Forest in the dry season (February) and in the Cocoa Crop and King Grass Crop in the wet season (August). Hobo S-THB-M8000 sensors were used for temperature (accuracy ±0,2°C) and relative humidity (accuracy ±2,5%) and the Hobo S-LIB-MOO3 sensor for solar radiation (irradiance) (accuracy ±10 W/m^2^). The measurements were taken 30 cm from the ground, every 30 seconds in the dry season and every minute in the wet season. In the dry season, the variables were measured the morning (9.30 am to 12 pm) of February 8^th^, 2019, in the cacao plantation, the afternoon (2.30 to 4.30 pm) of February 8^th^ and 9^th^ in the Star Grass paddock, and all day (9.30 am to 4.30 pm) on February 10^th^ in the Riparian Forest. In the wet season, on August 16^th^, in the cocoa plantation and in King Grass crop on August 17^th^, the same three variables were measured in the morning and/or the afternoon. The three climatic variables were measured in the wet season at 30 cm above the ground and only for cocoa crop habitat within the leaf litter (a refuge usually frequented by ticks).

## Results

### Morfological and molecular identification of *A*. *mixtum*

All collected adult stage samples were morphologically identified as *A*. *mixtum* (see S1 and S2 Figs). Selected samples of immature stages were morphologically identified as *Amblyomma* sp.

The molecular identification of *A*. *mixtum* was successful from eleven selected DNA samples (V7, V4, N3, L3, P44, P47, P77, P82, P140, P145, and P154) out of several *A*. *mixtum* DNA amplicons (see S4 Appendix), as we obtained their correspondant GenBank accession numbers: MZ959364.1 for V7, MZ059363.1 for V4, MZ959366.1 for N3, and MZ959365.1 for L3 (16S rRNA gene); MZ959406.1 for V7, MZ959405.1 for V4, MZ959408.1 for N3, MZ959407.1 for L3, OM757891.1 for P47, OM757892.1 for P77, OM757893.1 for P82, OM757894.1 for P140, OM757895.1 for P145, and OM757896.1 for P154 (COI gene); and MZ962661.1 for V4, MZ962662.1 for L3, OM780030.1 for P44, OM780031.1 for P47, OM780032.1 for P77, OM780033.1 for P82, OM780034.1 for P140, OM780035.1 for P145, and OM780036.1 for P154 (ITS-2 gene).

**Fig 3:**
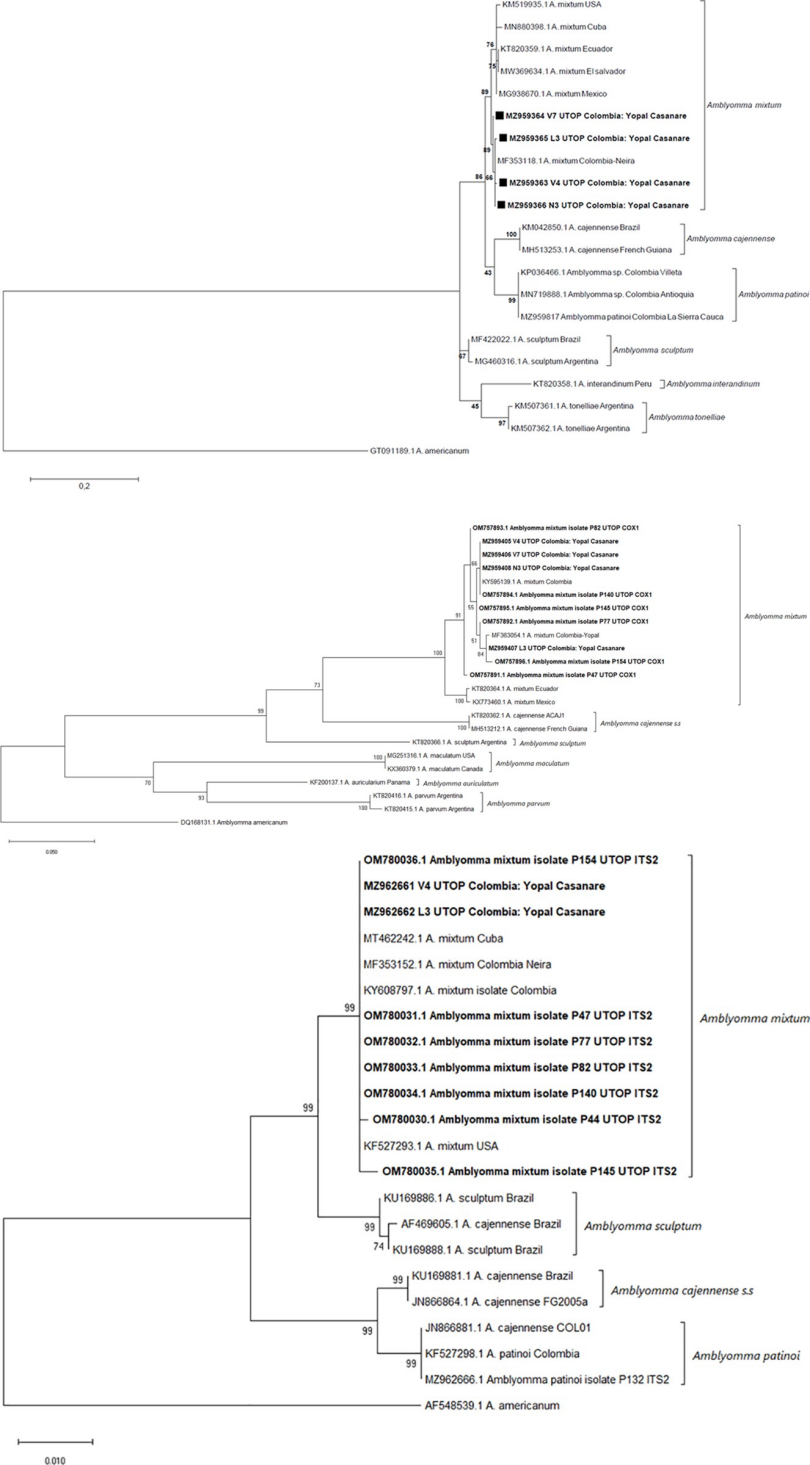
Phylogenetic trees for the 16S rRNA and COI mitochondrial genes and the ITS-2 nuclear gene of *A*. *mixtum*. Phylogenetic analysis using (A) the large subunit ribosomal RNA (16S-rRNA) gene sequences, (B) the Cytochrome C Oxidase subunit I (COX-1) gene sequences, and (C) the Internal Transcribed Spacer 2 (ITS-2) gene sequences of *Amblyomma mixtum* tick specimens collected in our study (in bold) and *A*. *mixtum* sequences from GenBank. Numbers at nodes are bootstrap support values. The correspondant gene sequence of *Amblyomma americanum* was used as the outgroup.

For the 16S rRNA gene (Fig 3A), the phylogenetic analysis shows two clusters (Boostrap support: 89%) visualized between V7, V4, N3, and L3 samples with a Colombian *A*. *mixtum* sequence previously deposited in the GenBank (MF353118.1) with a 98.19% identity for V7, V4 and L3, and 98.45% identity for N3. In the case of the COI gene (Fig 3B), one large cluster is formed (Boostrap support: 94%) between our samples and two *A*. *mixtum* sequences available in the GenBank with 100% identity for KY595139.1, between 99.20% (V4, V7, and N3) and 99.84% identity (L3) with MF363054.1, and between 99.07% (P47) to 99.69% (P154) identity with MT549814.1. Finally, the larger cluster (Boostrap support: 99%) was obtained with the ITS-2 gene (Fig 3C) with four *A*. *mixtum* GenBak sequences from Cuba (MT462242.1, between 100 and 99.30% identity for V4, N3, P44, P47, P77, P82, P140, and P154), Colombia (MF353152.1, 100% identity for V4 and between 99.30 to 99.90% to N3, P44, P47, P77, P82, P140, P145, and P154; KY608797.1, 100 and 99.35% identity for V4 and N3 respectively), and USA (KF527293.1).

In addition, several academic activities with free-living and parasitic stages of ticks have been carried out in the Matepantano farm for about six years, recording only adults of *A*. *mixtum* by morphological identification (EB, personal communication). In fact, in a previous study lead by EB (coauthor) [86], *Amblyomma* specimens were collected from both domestic animals and environment of several farms (including the Matepantano farm) in Yopal (Casanare, Colombia) and only *A*. *mixtum* was found by morphological and conventional Polymerase Chain Reaction coupled to sequencing for the Internal Transcribed Spacer 2 (ITS2) and the Cytochrome C Oxydase subunit I (COI) genes. Clearly, ticks from wild animals have not been recorded in the area within the last years, but several wild animal species have been seen moving thorugh the habitats by the locals (see two sections below). We observed capybaras near the cocoa crop during samplings. Also, EB’s team collected ticks from farms in Villeta (Cundinamarca, Colombia) and found only *A*. *patinoi* using the same tools [86]. There is an approximate distance of 430 km between Yopal (lowlands of Orinoquia region) and Villeta (850 m.a.s.l.), separated by part of the Andean mountains. The identification of *A*. *mixtum* ticks in the Matepantano farm concides with the findings of a previous study in the Yopal region [41].

### Overview of the relative abundance of stages of *A*. *mixtum*

Nymphs (59.7%; 4,021/6,733) dominated the relative abundance of *A*. *mixtum* in the dry season (February) for all selected habitats, larvae had 37.6% (2,533/6,733), and adults a low 2.7% (179/6,733). Meanwhile, in the wet season (August), the captured population decreased by one order of magnitude. It was dominated by adults with 61.7% (164/266) in three of the four habitats, except the Star Grass paddock, followed by larvae with 30.1% (80/266), and nymphs with 8.3% (22/266) (Table 2).

**Table 2:** Number of collected and identified *A*. *mixtum* specimens according to collection method, season, habitat type, and stage.

Collection method	Capture fraction in dry season (*in wet season*)	Habitat	Larvae in dry season (*in wet season*)	Nymphs in dry season (*in wet season*)	Females in dry season (*in wet season*)	Males in dry season (*in wet season*)	TOTAL in dry season (*in wet season*)
**CO**_**2**_ **traps**	7/7 (*2/8*)	Star Grass Paddock	205 (*0*)	431 (*10*)	17 (*12*)	3 (*10*)	**656 (*32*)**
	5/5 (*6/6*)	King Grass Crop	212 (*0*)	1,882 (*4*)	22 (*28*)	52 (*27*)	**2,168 (*59*)**
	8/8 (*11/12*)	Riparian Forest	532 (*0*)	757 (*5*)	39 (*18*)	24 (*26*)	**1,352 (*49*)**
	4/4 (*5/5*)	Cocoa Crop	61 (*0*)	729 (*0*)	10 (*13*)	5 (*22*)	**805 (*35*)**
**Subtotal**			**1,010 (*0*)**	**3,799 (*19*)**	**88 (*71*)**	**84 (*85*)**	**4,981 (*175*)**
**Cloth dragging by transects**	7/7 (*2/5*)	Star Grass Paddock	198 (*67*)	37 (*0*)	0 (*0*)	0 (*0*)	**235 (*67*)**
	6/6 (*2/7*)	King Grass Crop	496 (*13*)	74 (*0*)	0 (*1*)	0 (*0*)	**570 (*14*)**
	4/4 (*3/4*)	Riparian Forest	214 (*0*)	38 (*3*)	2 (*4*)	0 (*1*)	**254 (*8*)**
	5/5 (*2/6*)	Cocoa Crop	615 (*0*)	73 (*0*)	4 (*1*)	1 (*1*)	**693 (*2*)**
**Subtotal**			**1,523 (*80*)**	**222 (*3*)**	**6 (*6*)**	**1 (*2*)**	**1,752 (*91*)**
**TOTAL**			**2,533 (*80*)**	**4,021 (*22*)**	**94 (*77*)**	**85 (*87*)**	**6,733 (*266*)**

**Fig 4:**
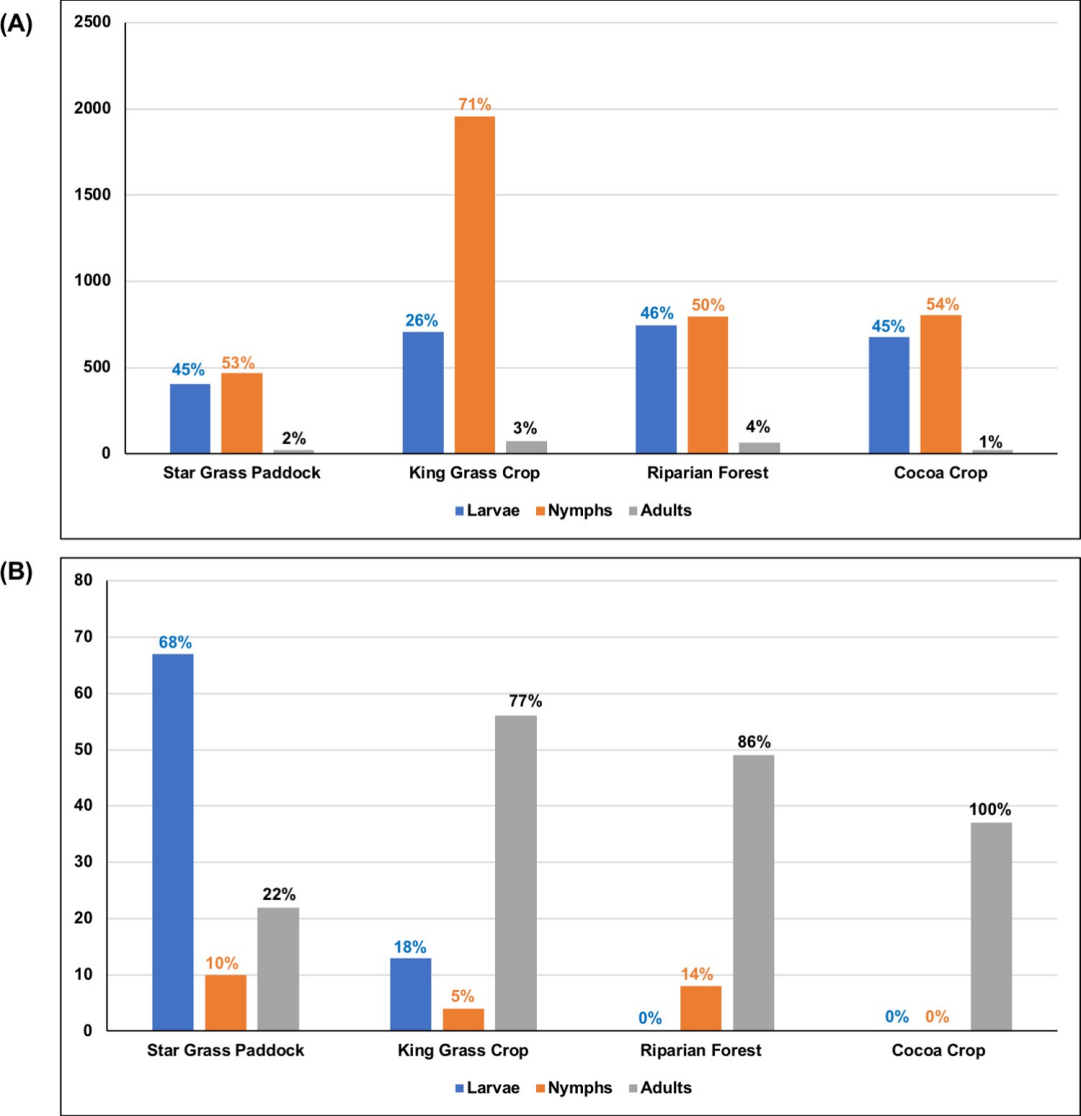
Abundance of *A*. *mixtum* in every sampled habitat according to the season in the Matepantano farm. (A). February of 2019 (dry season). (B). August of 2019 (wet season).

All the three *A*. *mixtum*’s stages (larvae, nymphs, and adults) used the four habitats with different proportions between the two periods of the year studied (Fig 4A and 4B and Table 2). In the dry season, 40% (2,738/6,733) of *A*. *mixtum* ticks were found in the King Grass Crop, 23.9% (1,606/6,733) in the Riparian Forest, 22.2% (1,498/6,733) in the Cocoa Crop, and 13.2% (891/6,733) in the Star Grass Paddock (Table 2). In the wet season, 37.2% (99/266) of the ticks used the Star Grass Paddock, followed by 27.4% (73/266) in the King Grass Crop, 21.4% (57/266) in the Riparian Forest, and 13.9% (37/266) in the Cocoa Crop (Table 2).

**Fig 5:**
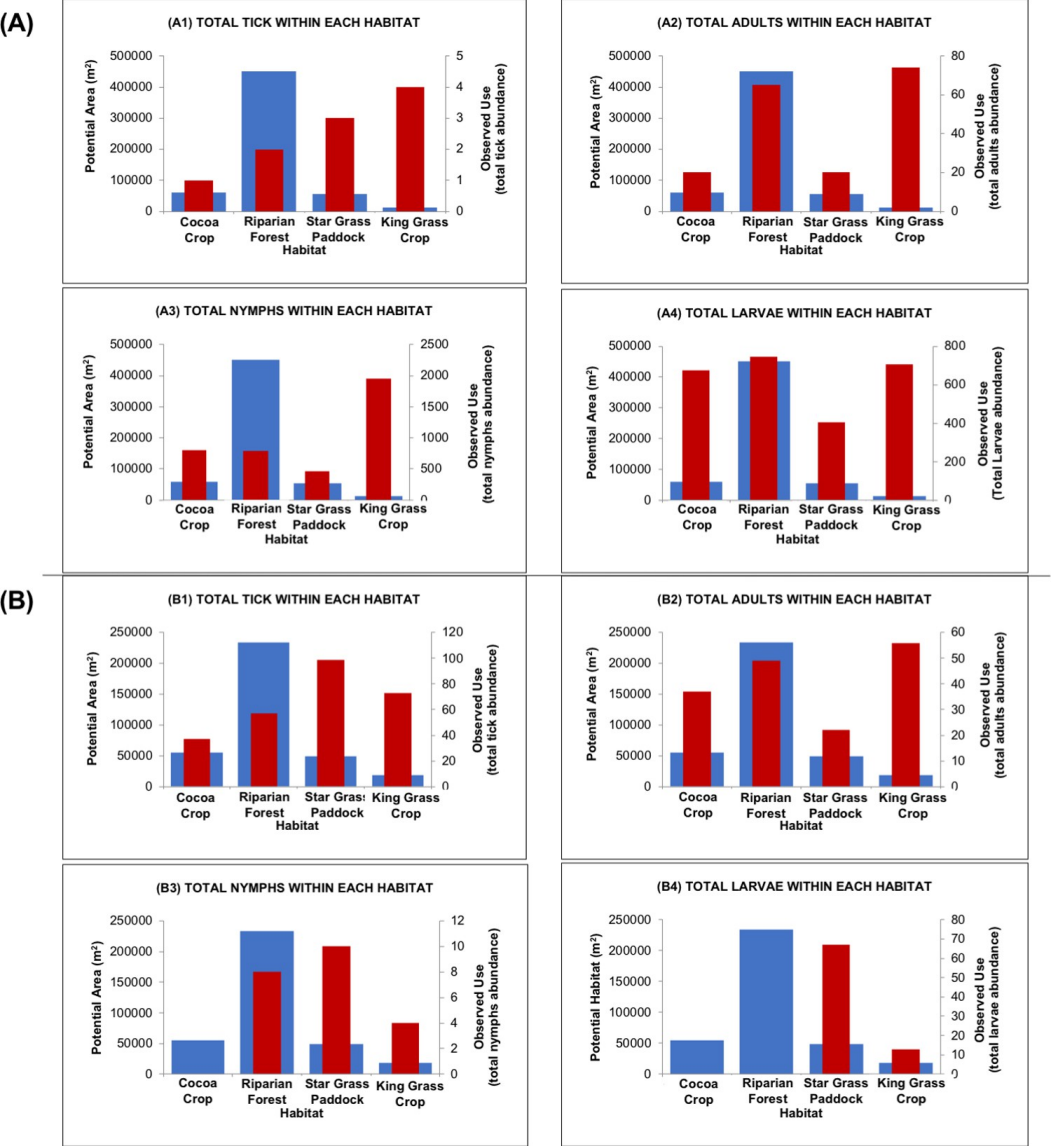
Trend graphs to infer the use and habitat preference of *A*. *mixtum*. These plots relate tick total abundance and tick stage abundance of both dry and wet seasons, (as an equivalent of observed use of each habitat resources) (red bar ∎; frequency on the y’-axis on the right) and the potential or available area of the habitat that *A*. *mixtum* might use (blue bar ∎; frequency on the y-axis on the left) at the Matepanano farm. Thus, the higher the red bar and the lower the blue bar, the greater the habitat preference. When the red bar barely exceeds or equals the blue bar, the relationship corresponds to habitat usage. When the blue bar far surpasses the red bar, there is avoidance of such a habitat (see tables 3–5). (A). Dry season. (A1) The mathematical index points out that *A*. *mixtum* uses all four habitats. In addition, *A*. *mixtum* population (stage independent) uses three habitats and shows preference for Star Grass Paddock and King Grass Crop, while it avoids the Riparian Forest habitat. (A2) Adults of *A*. *mixtum* prefer King Grass Crop, while they use the remainder habitats. (A3) Nymphs of *A*. *mixtum* prefer King Grass Crop and Cocoa Crop; they use Star Grass Paddock, but they avoid the Riparian Forest habitat. (A4) Larvae of *A*. *mixtum* prefers three habitats (King Grass Crop, Cocoa Crop, and Star Grass Paddock), while they only use the Riparian Forest habitat. (B). Wet season. (B1) *A*. *mixtum* population (stage independent) prefers King Grass Crop and Star Grass Paddock; it uses Cocoa Crop and avoids Riparian Forest. (B2) Adults of *A*. *mixtum* prefer King Grass Crop and Cacao Crop, while they use Star Grass Paddock and Riparian Forest. (B3) Nymphs of *A*. *mixtum* uses Cocoa Crop; they prefer Star Grass Paddock and King Grass Crop, and they avoid the Riparian Forest habitat. (B4) Larvae of *A*. *mixtum* only uses two out of four habitats, preferring the Star Grass Paddock, and using the King Grass Crop.

In the dry season, the three stages of *A*. *mixtum* used all the habitats (Figs 4A and 5A, and Tables 2–5). Only the adult stage of *A*. *mixtum* used all the habitats in both the dry and wet seasons. Remarkably, the adults’ absolute abundance remained relatively similar in both seasons. In the wet season, the nymphs did not use the Cocoa Crop habitat, and the larvae were absent in the Riparian Forest and Cocoa Crop habitats (Figs 4B and 5B, and Tables 2–5). *A*. *mixtum*’s gender ratio for the total adults in both periods (females:males with 171:172) was 0.994.

**Table 3:** Calculations of potential area and *A*. *mixtum* abundance within each habitat to obtain the observed use.

	Cocoa Crop	Riparian Forest	Star Grass Paddock	King Grass Crop	Total	Valor Chi (G-test)	Valor p (G-test)	Valor p (Chi-test)	Est. Error	n*pi>5 (Cherry test)	n*(1-pi)>5 (Cherry test)
**Potential Area (m** ^**2**^ **)**	**59,940**	**450,552**	**54,968**	**13,245**	**578,705**						
**Total ticks** ^**a**^	**1,498**	**1,606**	**891**	**2,738**	**6,733**	14,838.4	<0.001	<0.001	<0.001	Yes	Yes
Total adults^**a**^	20	65	20	74	179	338.7	<0.001	<0.001	0.0180	Yes	Yes
Total nymphs^a^^**a**^	802	795	468	1,956	4,021	11,019.2	<0.001	<0.001	0.0010	Yes	Yes
Total larvae^**a**^	676	746	403	708	2,533	3,788.5	<0.001	<0.001	0.0010	Yes	Yes
**Total ticks** ^**b**^	**37**	**57**	**99**	**73**	**266**	309.1	<0.001	<0.001	0.0070	Yes	Yes
Total adults^**b**^	37	49	22	56	164	163.5	<0.001	<0.001	0.0130	Yes	Yes
Total nymphs^**b**^	0	8	10	4	22	24.7	<0.001	<0.001	0.0780	No	Yes
Total larvae^**b**^	0	0	67	13	80	273.0	<0.001	<0.001	0.0200	No	Yes

^a^Ticks collected in the dry season

^b^ticks collected in the wet season.

**Table 4:** Calculations of the best five habitat preferences indices for *A*. *mixtum* in the Matepantano Farm by using HaviStat 2.4^®^.

HABITAT PREFERENCE INDEX	HABITAT				NOTES
** Duncan Index** [73]	**Cocoa Crop in dry season (*in wet season*)**	**Riparian Forest in dry season (*in wet season*)**	**Star Grass Paddock in dry season (*in wet season*)**	**King Grass Crop in dry season (*in wet season*)**	**Range:** 0 to +Inf. **If > 0.3 = Preference** If < 0.3 = No Preference — = absence of individuals
**Total tick abundance**	**0.5** (*0*.*3*)	0.1 (*0*.*1*)	**0.4 (*0*.*6*)**	**1.3 (*0*.*8*)**	
**Total adults**	0.3 (***0*.*4***)	0.2 (*0*.*2*)	0.3 (*0*.*3*)	**1.3 (*0*.*9*)**	
**Total nymph**	**0.5** (—)	0.1 (*0*.*2*)	**0.4 (*0*.*6*)**	**1.3 (*0*.*7*)**	
**Total larvae**	**0.6** (—)	0.1 (—)	**0.4 (*0*.*9*)**	**1.1 (*0*.*6*)**	
** Ivlev’s Electivity Index** [74]					**Range:** -1 to +1 **If > 0 = Preference** If < 0 = No Preference
**Total tick abundance**	**0.4** (*-0*.*1*)	-0.5 (*-0*.*5*)	**0.2 (*0*.*5*)**	**0.9 (*0*.*7*)**	
**Total adults**	0.0 (***0*.*2***)	-0.4 (*-0*.*4*)	**0.1** (*0*.*0*)	**0.9 (*0*.*7*)**	
**Total nymph**	**0.3** (—)	-0.6 (*-0*.*3*)	**0.1 (*0*.*5*)**	**0.9 (*0*.*6*)**	
**Total larvae**	**0.4** (—)	-0.5 (—)	**0.3 (*0*.*7*)**	**0.8 (*0*.*5*)**	
** Bailey Confidence Intervals** [75]					**If upper Interval< Species Use = Preference** If Lower interval > Species Use = No Preference If Lower Interval< Species Use < Upper Interval = Use — = absence of individuals
**Total tick abundance**	**697.4** (*41*.*1*)	5,242.0 (*175*.*0*)	**639.5 (*36*.*4*)**	**154.1 (*13*.*5*)**	
**Total adults**	18.5 (*25*.*4*)	139.4 (*107*.*9*)	17.0 (*22*.*5*)	**4.1 (*8*.*3*)**	
**Total nymph**	**416.5** (—)	3,130.6 (*14*.*5*)	**381.9 (*3*.*0*)**	**92.0** (*1*.*1*)	
**Total larvae**	**262.4** (—)	1,972.1 (—)	**240.6 (*11*.*0*)**	**58.0 (*4*.*1*)**	
** Alpha Index** [76,77] **(Constant Resources** [78]**)**					**Range:** 0 to +1 **If > 1/(#Indep. Var.) = Preference** If < 1/(#Indep. Var.) = No Prefererence 1/(#Indep. Var.) = 0.25 — = absence of individuals
**Total tick abundance**	0.1 (*0*.*1*)	0.0 (*0*.*0*)	0.1 (***0*.*3***)	**0.8 (*0*.*6*)**	
**Total adults**	0.1 (*0*.*2*)	0.0 (*0*.*0*)	0.1 (*0*.*1*)	**0.9 (*0*.*7*)**	
**Total nymph**	0.1 (—)	0.0 (*0*.*1*)	0.0 (***0*.*4***)	**0.9 (*0*.*5*)**	
**Total larvae**	0.2 (—)	0.0 (—)	0.1 (***0*.*7***)	**0.7 (*0*.*3*)**	
** Interpretation of II** [79]					**Range:** -1 to +1 If -1 < Index Value < -0.5 = Strong Avoidance If -0.49 < Index Value < -0.26 = Moderate Avoidance If -0.25 < Index Value < 0.25 = Indiference If 0.26 < Index Value < 0.49 = Neutral Selection **If 0.50 < Index Value < 1 = Strong Selection** — = absence of individuals
**Total tick abundance**	0.4 (*-0*.*1*)	-0.8 (*-0*.*8*)	0.2 (***0*.*6***)	**0.9 (*0*.*8*)**	
**Total adults**	0.0 (*0*.*2*)	-0.7 (*-0*.*6*)	0.1 (*0*.*0*)	**0.9 (*0*.*8*)**	
**Total nymph**	0.4 (—)	-0.9 (*-0*.*5*)	0.1 (***0*.*7***)	**1.0 (*0*.*6*)**	
**Total larvae**	0.5 (—)	-0.8 (—)	0.3 (***0*.*9***)	**0.9 (*0*.*6*)**	

**NOTE:** Potential area (m^2^) for each habitat and tick abundance were used from Table 3 for each index calculation.

**Table 5:** Calculations of the best five niche amplitude indices for *A*. *mixtum* within its habitats in the Matepantano Farm by using HaviStat 2.4^®^.

TICK STAGE ABUNDANCE	TICKS COLLECTED PER HABITAT				NICHE AMPLITUDE INDEX	NOTES
	**Cocoa Crop in dry season (*in wet season*)**	**Riparian Forest in dry season (*in wet season*)**	**Star Grass Paddock in dry season (*in wet season*)**	**King Grass Crop in dry season (*in wet season*)**	**Standardized Shannon Index** [81] **in dry season (*in wet season*)**	**Bolded value** = max. amplitude Underlined value = min. amplitude **Range:** 0 to +1 **0** = minimum niche amplitude **+1** = maximum niche amplitude — = absence of individuals
**Total**	1,498 (*37*)	1,606 (*57*)	891 (*99*)	2,738 (*73*)	0.94 (***0*.*96*)**	
**Total adults**	20 (*37*)	65 (*49*)	20 (*22*)	74 (*56*)	0.88 (***0*.*96***)	
**Total nymphs**	802 (—)	795 (*8*)	468 (*10*)	1,956 (*4*)	0.90 (*0*.*75*)	
**Total larvae**	676 (—)	746 (—)	403 (*67*)	708 (*13*)	**0.98 (** ***0*.*32*** **)**	
					**Ivlev’s Electivity Index** [74] **in dry season (*in wet season*)**	**Range:** 0 to +infinite **0** = maximum niche amplitude **+infinite** = minimum niche amplitude — = absence of individuals
**Total**	1,498 (*37*)	1,606 (*57*)	891 (*99*)	2,738 (*73*)	1.95 (*1*.*71*)	
**Total adults**	20 (*37*)	65 (*49*)	20 (*22*)	74 (*56*)	**1.38** (***1*.*31***)	
**Total nymphs**	802 (—)	795 (*8*)	468 (*10*)	1,956 (*4*)	1.92 (*2*.*39*)	
**Total larvae**	676 (—)	746 (—)	403 (*67*)	708 (*13*)	1.99 (*3*.*24*)	
** **					**Levins Index** [82] **in dry season** **(*in wet season*)**	**Range:** 0 to +2 **0** = minimum niche amplitude **+2** = maximum niche amplitude — = absence of individuals
**Total**	1,498 (*37*)	1,606 (*57*)	891 (*99*)	2,738 (*73*)	1.31 (***1*.*33***)	
**Total adults**	20 (*37*)	65 (*49*)	20 (*22*)	74 (*56*)	1.22 (***1*.*33***)	
**Total nymphs**	802 (—)	795 (*8*)	468 (*10*)	1,956 (*4*)	1.24 (*1*.*04*)	
**Total larvae**	676 (—)	746 (—)	403 (*67*)	708 (*13*)	**1.36** (*0*.*44*)	
** **					**Standardized Levins Index** [82] **in dry season (*in wet season*)**	**Range:** 0 to +1 **0** = minimum niche amplitude **+1** = maximum niche amplitude — = absence of individuals
**Total**	1,498 (*37*)	1,606 (*57*)	891 (*99*)	2,738 (*73*)	0.82 (*0*.*86*)	
**Total adults**	20 (*37*)	65 (*49*)	20 (*22*)	74 (*56*)	0.68 (***0*.*88***)	
**Total nymphs**	802 (—)	795 (*8*)	468 (*10*)	1,956 (*4*)	*0*.*68* (*0*.*56*)	
**Total larvae**	676 (—)	746 (—)	403 (*67*)	708 (*13*)	**0.94** (*0*.*12*)	
** **					**Modified Standardized Levins Index** [82] **in dry season(*in wet season*)**	**Range:** (1/Independent Variable) to +1 **(1/Independent Variable)** = minimum **+1** = maximum niche amplitude — = absence of individuals
**Total**	1,498 (*37*)	1,606 (*57*)	891 (*99*)	2,738 (*73*)	0.86 (*0*.*90*)	
**Total adults**	20 (*37*)	65 (*49*)	20 (*22*)	74 (*56*)	0.76 (***0*.*91***)	
**Total nymphs**	802 (—)	795 (*8*)	468 (*10*)	1,956 (*4*)	0.76 (*0*.*67*)	
**Total larvae**	676 (—)	746 (—)	403 (*67*)	708 (*13*)	**0.96** (*0*.*34*)	

**NOTE:** Potential area (m^2^) for each habitat and tick abundance were used from Table 3 for each index calculation.

In total, 2.8 times more ticks were caught with CO_2_ traps (4,981) than with white flannel transects (1,752) in the dry season. In the rainy season, the CO_2_ traps’ effectiveness for tick collection decreased 1.9 times (175 individuals) compared to the transects (91 individuals). The flannel dragging method in the pre-determined transects was most effective in capturing the larvae in both dry and wet seasons compared to the remnant stages (Table 2). Up to 17 times more nymphs and 25 times more adults were collected using the CO_2_ traps than in the transects in the dry season. Similarly, six times more nymphs and 20 times more adults were captured in the wet season using CO_2_ traps than flannel dragging. In all habitats, CO_2_ traps were more effective in collecting ticks in dry season. In the wet season, they were effective in 75% of the habitats. The transects only outperformed the traps in capturing ticks in the Star Grass paddock habitat in the wet season (Table 2).

### Habitat use and preference

The Cherry Test [80] indicated that, in both dry and wet seasons, the total sample of *A*. *mixtum* ticks captured was sufficient to infer on the use and preference (Table 3). Duncan’s Preference Indexes [73], of Ivlev’s electivity [74], Bailey’s confidence interval [75], Alpha [76,77] (Constant Resources [78]), and Interpretation of II [79,83] indicated that the *A*. *mixtum* population used all four habitats (Fig 5 and Table 4). Moreover, most of these indices showed *A*. *mixtum*’s preference for three of the four habitats (King Grass Crop, Cocoa Crop, and Star Grass Paddock).

*A*. *mixtum* uses, but does not prefer, the Riparian Forest habitat. This result was consistent for nymphs, larvae, and adults; this was corroborated by the interpretation of Index II that indicated a strong avoidance of this habitat (Table 5). Although adults also preferred the King Grass Crop habitat in both seasons, according to all the selected indices, they used, but did not prefer, the cocoa plantation and Star Grass Paddock in the dry season according to four of the five indices (Table 4). In the wet season, the concordance of non-preference of adults decreased between indices concerning the Cocoa Crop habitat, but remains regarding the Star Grass Paddock. The interpretation index of II shows a strong avoidance of the Riparian Forest habitat by the adults (Table 4). In the case of larvae and nymphs, in the dry season, the concordance of preference between indices is not complete either. Larvae and nymphs prefer three of the four habitats according to three of the five selected indices. In the wet season, the five indices indicated preference of the immature stages for the habitats Star Grass Paddock and King Grass Crop (Table 4).

### Niche width

In the dry season, the niche amplitude was maximum for the larvae in four of the five indices, as the value of Ivlev’s electivity index was the lowest. In general, the rates of niche width obtained in this part of the year are considered high for all free-living stages of *A*. *mixtum*, as well as the population as a whole, since the values obtained tend towards the maximum amplitude (Table 5) [74,81,82,84]. Interestingly, during the wet season, the five selected indices coincided in a maximum niche amplitude for the adult stage (Table 5). In both the dry and wet seasons, the survival rate of adults was similar (Table 2) and may indicate that adults are present at different times throughout the year. In contrast, the population of larvae and nymphs almost succumbed in the studied wet season (absent from the Cocoa Crop habitat samples), reducing their numbers by one order of magnitude when compared to their collection in the studied dry season. Larvae were also not found in the Riparian Forest habitat (Fig 4 and Table 2). Remarkably, the larvae in the studied wet season obtained the lowest niche amplitude in all the selected indices (Table 5).

### Climate of the region in dry and wet seasons

Wide seasonal variation of ambient temperature was observed in the region of up to 12°C on average between both the dry and wet seasons (Table 6), and an absolute range of 26°C at the local weather station located in the Cocoa Plantation habitat (Table 7). The temperature variation in the dry season at the Matepantano farm is greater (from 18°C to 37°C) when compared to the wet season (minimum of 19°C and maximum of 33.6°C). Total precipitation in the wet season (August) was one order of magnitude higher than in the dry season (Table 7). The average relative humidity varied by 20% between February and August, both in the regional (61 and 81%) and local (68 and 88%) climate seasons, reaching minimum values of 30% in the dry season (Tables 6 and 7).

**Table 6:** Multiannual mean range for several climatic variables recorded for 29 years (1975–2004) in two weather stations located at the Yopal municipality (Casanare, Colombia) (regional scale).

Variable	Range of the multiannual mean^a^	Observations
Temperature (°C)	24–28	The driest period was from November to March. Maximum and minimum values of the multiannual mean temperature during the 29 years period were 33.7 and 30.1°C for February, and 23.8 and 21.7°C for August.
Total precipitation (mm)	2,500–3,000	A unimodal rainfall cycle with a peak from April to October. The maximum precipitation/day reached 200 mm in just one day. The multiannual mean precipitation was 16 mm for February and 97 mm for August.
Evaporation (mm/year)	1,300–1,500	Almost a half of the rainfall was evaporated.
Potential evapotranspiration (mm/year)	1,200–1,400	
Relative humidity (%)	75–80	Perhumid climatic type (Thornthwaite classification Limit 7/9). Multiannual mean relative humidity between 1981 to 2010 was 61% for February and 81% for August.
Daily hours of sunligth	5–6	
Daily solar irradiation (KW/h/m^2^)	4.5–5	
Ultraviolet radiation (IUV)	7–8	
Soil net water (mm)	1,000–2,000	Soil drought was common between the November to January period.
Water excess in the annual extreme (decades)	15–20	
Water deficit in the annual extreme (decades)	5–10	
Wind speed (m/s)	3–4	The prevailing wind direction at the study site was East-West.

^a^Data provided by IDEAM (IDEAM, [Climatic Atlas of Colombia, Interactive version, Bogotá, D.C., 2015, text in Spanish]**ISBN: Volume 1: 978-958-8067-73-5**) corresponded to the annual mean with annotations on variable behavior each month. However, no access was obtained to original data to calculate maximum and minimum values of each variable during those 29 years. **NOTE:** According to the IDEAM, the agroclimatic zone value of the study site has been <0.6 within the last three decades.

**Table 7:** Multiannual mean of climatic variables recorded for seven years (2012–2019) in the Matepantano farm (local scale).

Variable^a^	Mean (SD)	Max.	Min.	Difference	Observations
Annual temperature (°C)	25.9(±3.5)	41.9	15.9	26.0	Maximum value was recorded in 2018, while the minimum value was recorded in 2015.
Temperatura (°C) in February (summer)	27.7(±4.1)	37.0	18.0	19.0	
Temperatura (°C) in August (winter)	25.2(±3.0)	33.6	19.0	14.6	
Total annual precipitation (mm)	1,559.8(±507.7)	2,251	619	1,632	Maximum precipitation (155 mm in one day) between July to November of 2016.
Total precipitation (mm) in February	23.7(±37.7)	105	0	105	
Total precipitation (mm) in August	366(±104.3)	481	193	288	It represents almost the third of the annual total precipitation.
Relative humidity (%)	83.4(±13.6)	100	30	70	
Relative humidity (%) in February	68.0(±14.5)	99	30	69	
Relative humidity (%) in August	88.3(±8.6)	100	50	50	
Dew point (°C)	22.2(±4.7)	27.2	18.2	9	
Solar irradiation (KW/m^2^)	221.7(±317.0)	1,447	0	1,447	
Solar energy -radiant light and heat- (Exajoules)	3.3(±6.3)	46.6	0	46.6	
Atmospheric pressure (milibar)	1,010(±2)				
Wind speed (m/s)	0.76(±0.36)	16.1	0	16.1	Often strong gusts of wind in the N-NE and E directions. The strongest gusts of wind between August to November. Only throughout the 2015 year, the wind direction was to the North.

^**a**^Data from the weather station located at the Cacao Crop habitat (N = 242,152 records).

**NOTE:** A milimeter of water equals the emptying of one (1) liter (L) of water in a 1 m^2^ surface of enclosed space (the water volume will have exactly 1 mm of thickness). So, the multiannual mean precipitation in February (summer) in the Matepantano farm was 23.7 L/m^2^ that is equal to 23.7 mm or around 2 cm of water above ground. By contrast, the multiannual mean precipitation in August (winter) was 366 L/m^2^ (366 mm or almost 36 cm of water above ground). This means around 15 times more ponding per m^2^ in August that in February.

### Microclimatic variables by habitat (dry and/or wet seasons)

#### Cocoa crop

**Fig 6:**
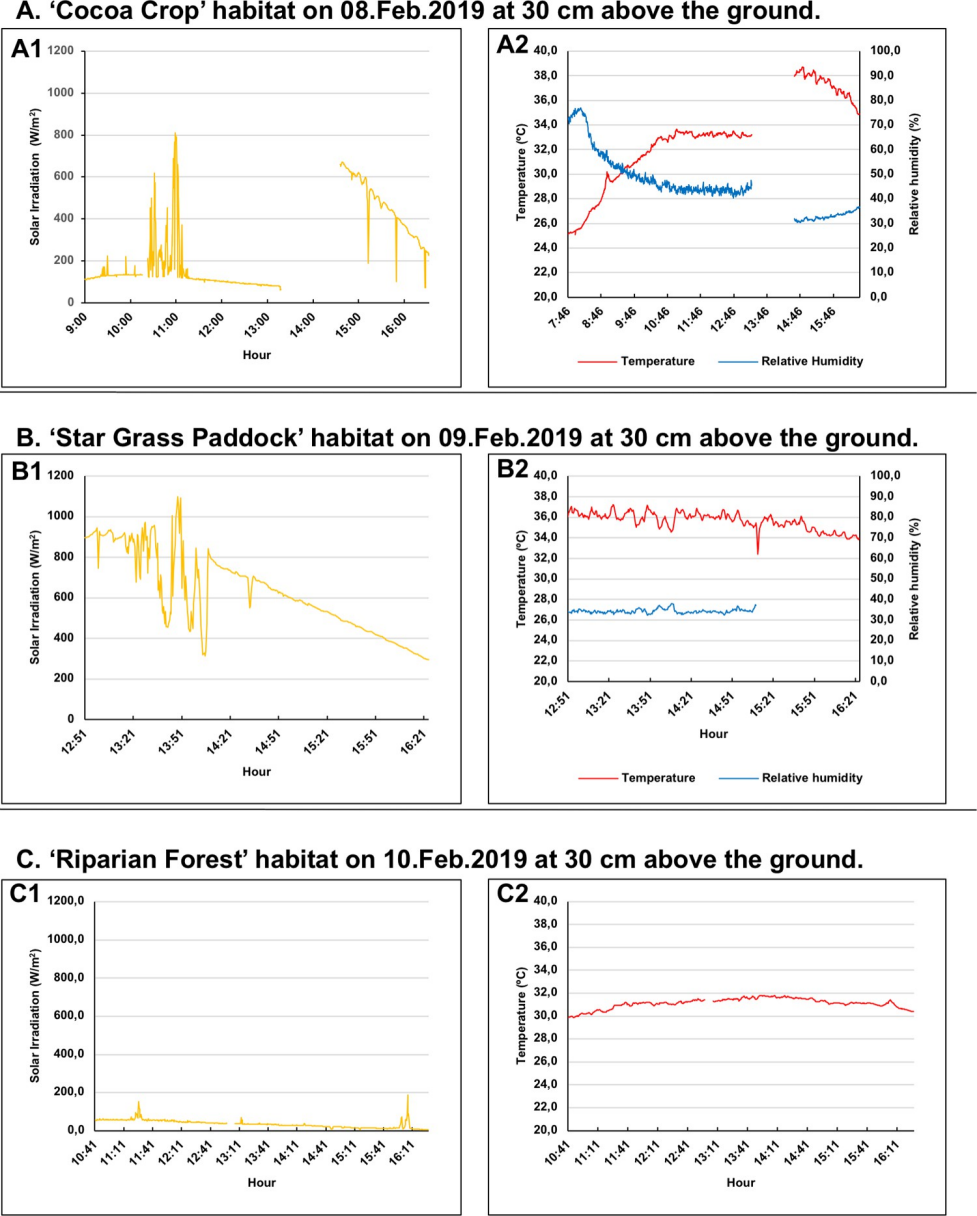
Plots of microclimatic variables that were measured *in situ* for three habitats in the dry season. (A). Cocoa Crop habitat. (A1). Solar irradiation values (W/m^2^) (N = 736; $\bar{\chi }$ = 243.5; SD = 189.1; Max = 810.6; Min = 59.4) according to the daytime hours. (A2). Temperature values (°C; red line) (N = 901; $\bar{\chi }$ = 32.7; SD = 3.6; Max = 38.7; Min = 25.1) and relative humidity (%; blue line) (N = 901; $\bar{\chi }$ = 45.9; SD = 11.6; Max = 76.9; Min = 30.4) according to the daytime hours. (B). Star Grass Paddock habitat. (B1). Solar irradiation values (W/m^2^) (N = 427; $\bar{\chi }$ = 629.1; SD = 199.6; Max = 1,096.9; Min = 295.6) according to the daytime hours. (B2). Temperature values (°C; red line) (N = 427; $\bar{\chi }$ = 35.6; SD = 0.8; Max = 37.2; Min = 32.4) and relative humidity (%; blue line) (N = 276; $\bar{\chi }$ = 34.4; SD = 1.0; Max = 38; Min = 32.3) according to the daytime hours. The relative humidity variable was not measured during the same period of time than temperature because of a damage in the respective sensor. (C). Riparian Forest habitat. (C1). Solar irradiation values (W/m^2^) (N = 677; $\bar{\chi }$ = 36; SD = 19.8; Max = 189.6; Min = 6.9) according to the daytime hours. (C2). Temperature values (°C) (N = 677; $\bar{\chi }$ = 31.1; SD = 0.4; Max = 31.8; Min = 29.9) according to the daytime hours. The relative humidity variable was not measured because of a damage in the respective sensor.

**Fig 7:**
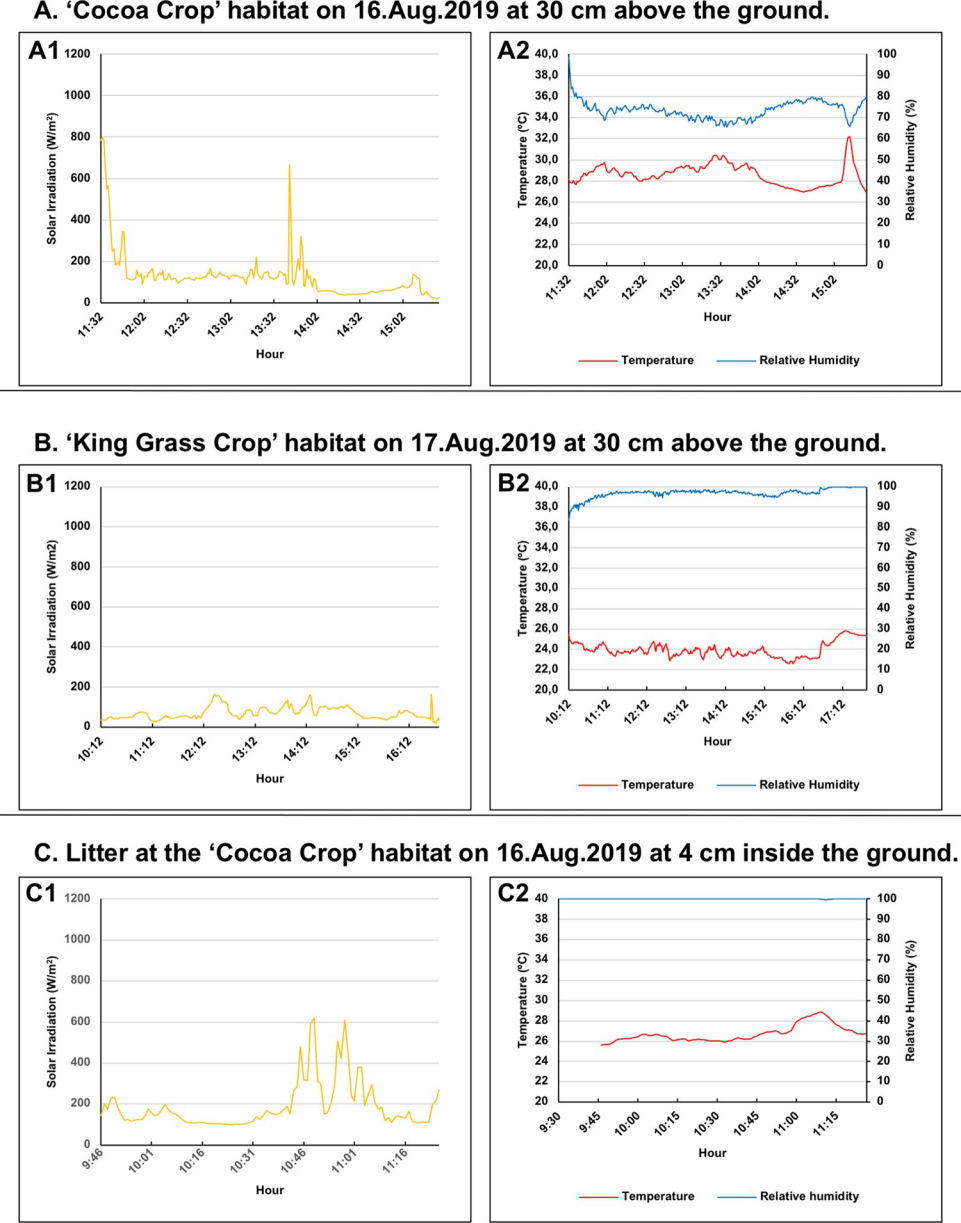
Plots of microclimatic variables that were measured *in situ* for two habitats in the wet season. (A). Cocoa Crop habitat. (A1). Solar irradiation values (W/m^2^) (N = 236; $\bar{\chi }$ = 126.4; SD = 118.3; Max = 798.1; Min = 19.4) according to the daytime hours. (A2). Temperature values (°C; red line) (N = 236; $\bar{\chi }$ = 28.6; SD = 1.0; Max = 32.2; Min = 27.0) and relative humidity (%; blue line) (N = 236; $\bar{\chi }$ = 73.6; SD = 4,3; Max = 98.6; Min = 65.4) according to the daytime hours. (B). King Grass Crop habitat. (B1). Solar irradiation values (W/m^2^) (N = 396; $\bar{\chi }$ = 70.4; SD = 30.2; Max = 164.4; Min = 19.4) according to the daytime hours. (B2). Temperature values (°C; red line) (N = 457; $\bar{\chi }$ = 23.9; SD = 0.7; Max = 25,8; Min = 22.6) and relative humidity (%; blue line) (N = 457; $\bar{\chi }$ = 96.9; SD = 2.3; Max = 100; Min = 83.6) according to the daytime hours. (C). Litter at the Cocoa Crop habitat. (C1). Solar irradiation values (W/m^2^) (N = 101; $\bar{\chi }$ = 188.7; SD = 114.1; Max = 619.4; Min = 99.4) according to the daytime hours. (C2). Temperature values (°C) (N = 101; $\bar{\chi }$ = 26,7; SD = 0,8; Max = 28,8; Min = 25,6) and relative humidity (%) (N = 117; $\bar{\chi }$ = 99.99; SD = 0.05; Max = 100; Min = 99.6) according to the daytime hours.

The average irradiance was almost half in the wet season than in the dry season (126 vs. 243 W/m^2^ [Figs 6 and 7]). The variation in temperature was also lower in the wet season (just 5°C) when compared to the dry season (13°C). Likewise, the variation in relative humidity was also lower in the wet season, with about 30% between the extreme values when compared to 47% in the dry season (Figs 6 and 7). Higher values of relative humidity early in the morning coincided with observations of increased tick activity and abundance in the dry ice traps at the time of capture.

#### Litter in cocoa crop

The variables solar irradiation, temperature, and humidity inside the litter, that is, between the soil and the litter, were taken in order to compare the litter (a place used as a refuge by ticks because its lower micro-environmental variation) with the Cocoa Crop microenvironment at 30 cm from the ground. Thus, the average temperature inside the leaf litter (7 cm deep) was two degrees lower than in the environment, and the variation between extreme values was narrower (Fig 7). The humidity was maximum within the litter, almost completely constant (100% for most of the period studied) (Fig 7). Although the measurements were not simultaneous between the litter and the microclimate at 30 cm from the ground, the solar irradiation remained below 200 W/m^2^ most of the time in both environments (Fig 7), indicating that sunlight reaches the leaf litter at a depth of 7 cm.

#### Star grass paddock

There was a difference of 800 W/m^2^ (max. 1.100 and min. 300 W/m^2^) in solar radiation in a sigle-day measure. The temperature remained high on average at 35°C, decreasing little in the afternoon hours. The relative humidity in the Star Grass Paddock was low in the dry season (34% on average). The variation between extreme values was also low (6%) (Fig 6).

#### Riparian forest

In this habitat, solar irradiation was very low, with an average of 36 W/m^2^ and a variation between extreme values of 180 W/m^2^ (Fig 6). This low penetration of sunlight, due to the high coverage of the canopy, maintained a relatively constant temperature with an average of 31°C, varying only 2°C. Unfortunately, due to damage to the humidity sensor, the respective values were not recorded that day.

#### King grass crop

Solar radiation was low with an average of 70 W/m^2^ and a difference of 145 W/m^2^ between the extreme values recorded (cloudy day), which can be explained by the height of the King Grass (about 2 m) and the dense vegetation. In this sense, the humidity was very high at 30 cm from the ground, between 83–100% with an average of 97% during that day (Fig 7). The range of temperature variation throughout the day was also narrow, with only 3°C and an average of 24°C.

The average solar radiation captured in the dry season in the habitat of Star Grass Paddock was 2.5 times higher than in the Cocoa Crop and more than 17 times higher than in the Riparian Forest. In the same sense, the average solar radiation measured in the Cocoa Crop in the wet season was 1.8 times higher than the King grass recorded values, while the litter had an average 1.35 times higher in relative humidity than the Cocoa Crop habitat. Therefore, the climatic and microclimatic data show that, within the Matepantano farm, *A*. *mixtum* may live in an environment with significant variations in a single day and between seasons.

### Potential hosts seen by locals

**Fig 8:**
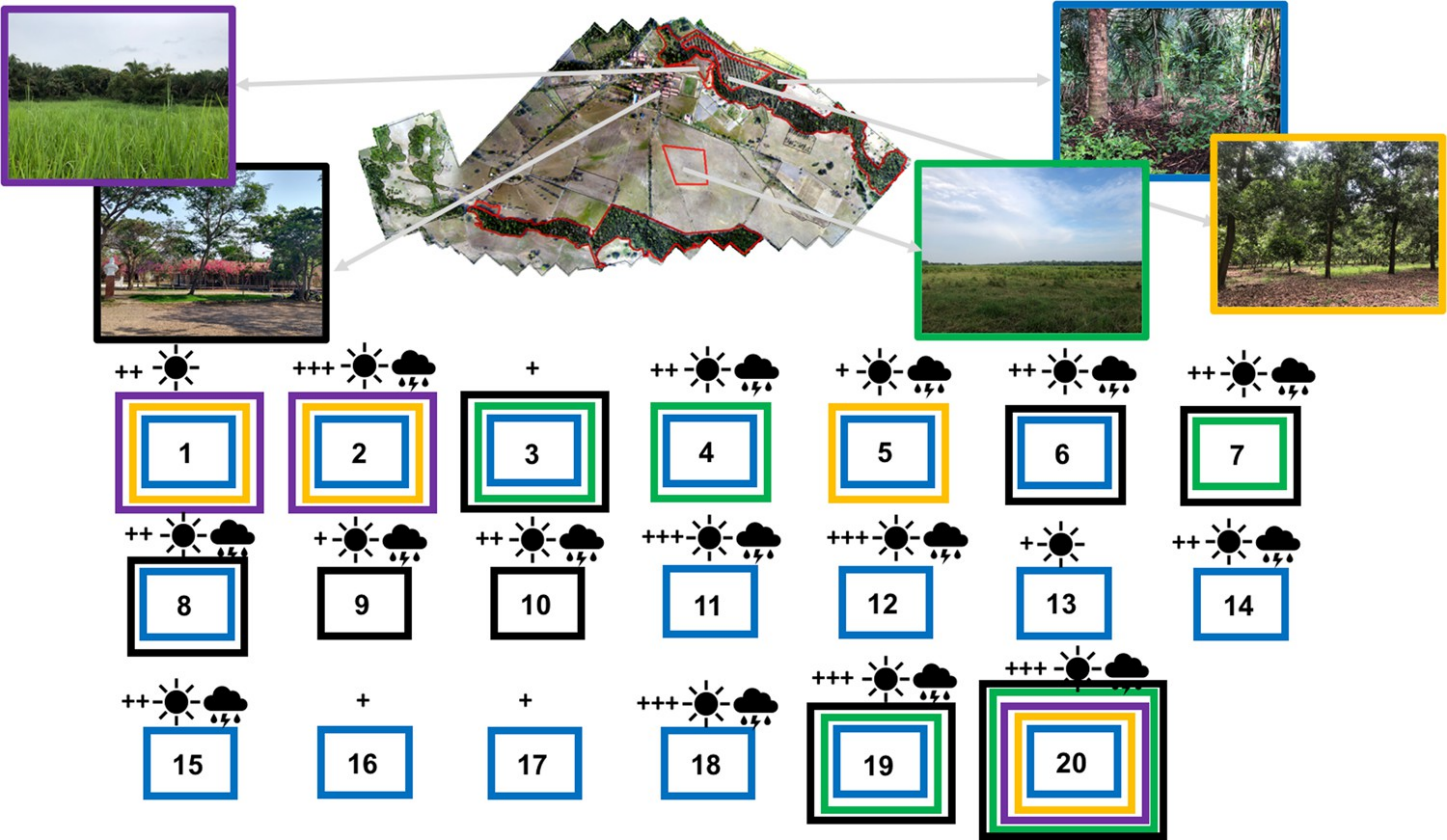
Potential host mammals of *A*. *mixtum* seen by people in the habitats of the Matepantano farm. Color of every box (blue = Riparian Forest; orange = Cocoa Crop; violet = King Grass Crop; green = Star Grass Paddock; black = administrative buildings; bedrooms, classroom, and restaurant) represent every habitat and other areas where the potential host has been seen by locals. Also, the sun symbol represents host presence in the dry season, while the rain clouds stands for host presence in the wet season. The ‘+’ symbol indicates the minimum perception of relative host abundance in the habitat, while the ‘+++’ represents the maximum perception. Note that host could have been seen in different habitats (several colored boxes). The animals species identified by locals based on photographs and common names have been identified by numbers and organized by rows from the left to the right and their common and scientific names (the last one between parentheses) are written as follows: 1) Armadillo (***Dasypus novemcinctus***); 2) capybara (***Hydrochoeris hydrochaeris***); 3) porcupine (*Coendou prehensilis*); 4) black agouti (*Dasyprocta fuliginosa*); 5) deer (*Odocoileus virginianus*); 6) flat-faced fruit-eating bat (*Artibeus planirostris*); 7) anteater (*Myrmecophaga tridactyla*); 8) Seba’s short-tailed bat (*Carollia perspicillata*); 9) fox (*Urocyon cinereoargenteus*); 10) opossum (*Didelphis marsupialis*); 11) tufted capuchin (*Cebus apella*); 12) Colombian red howler (*Alouatta seniculus*); 13) collared peccary (*Pecari tajacu*); 14) South American tapir (***Tapirus terrestris***); 15) southern tamandua (***Tamandua tetradactyla***); 16) puma (*Puma yagouaroundi*); 17) margay (*Leopardus wiedii*); 18) cat (*Felis catus*); 19) cow (***Bos taurus indicus***); 20) horse (***Equus ferus caballus***). Except for the last three species, all 17 animals seen by locals in the habitats are wild species. Both domestic and wild animals use to be around the administrative buildings. Bolded scientific names belong to *A*. *mixtum* hosts reported [30].

A total of 20 mammals were identified as potential hosts on the farm, seen by people moving through the four habitats compared (Fig 8). Eighty-five percent (17/20) of the animals were seen in Riparian Forest, 40% (8/20) in the area of administrative and residential buildings, 25% (5/20) in Star Grass Paddock, 20% (4/20) in Cocoa Crop, and 15% in King Grass Crop (3/20) (Fig 8). Only three of the 20 animals were classified as domestic, the rest were wild mammals. Armadillos, capybaras, horses, and cattle were identified by respondents as the species most frequently seen passing through the farm and connecting the four habitats. Six species of animals (three domestic and three wild) were the mammals most oftenly seen. Apart from the three species of domestic animals (cats, horses, and cows), 14 species of wild mammals in the dry season and 12 in the wet season were consistently observed by respondents. It is interesting to note that the dry season was the time with the highest tick capture in our study.

## Discussion

The literature regarding empirical data on *A*. *mixum* tolerance to different abiotic variables is still incipient. Even so, the available data about *A*. *mixtum* in Texas (USA), Mexico, and Panama indicate that the species, in the field as well as in the laboratory, can tolerate high and low relative humidity, as well as high and low temperature (ranges: 35–95% and 18-36°C; see S5 Table). The thermal tolerance reported for *A*. *mixtum* coincides with our observations, where the temperature fluctuates strongly between the two seasons of the year studied (the dry season in February/2019 vs. the wet season in August/2019). In the Yopal region, the average temperature has been between 21 and 33°C for three decades (Table 6) and specifically at the Matepantano farm, between 18 and 37°C, for the last seven years (Table 7). We found that *A*. *mixtum* achieved the maximum niche width in the dry season. In this way, we hypothesize that the studied population of *A*. *mixtum* would be favored by the wide temperature changes that occur at different time scales (e.g., daily and seasonal) in the habitats.

One model projected that *A*. *mixtum* in California finds no suitable habitat for establishing its populations for two related limitations, the low average temperature of 10°C associated with high altitude [50]. Additionally, *A*. *mixtum* was not found above 1,200 m.a.s.l. in Panama, in places with less than 15°C [34]. The thermal limit has also been corroborated, where 50% of the adults of *A*. *cajennense* s.l. (renowned *A*. *mixtum*) died after 180 minutes of exposure to -12.5°C [87], limiting its spatial distribution in latitudes higher than 30° [88]. From the above, it is inferred that the appropriate temperature in the habitats considered used, or preferred by *A*. *mixtum*, can vary widely within extreme values (10–15°C), favoring this species. *A*. *mixtum’s* altitudinal distribution in the foothills of the Eastern Colombian Mountains and its negative association with low temperatures, despite the presence of favorable hosts such as horses and cows, should be investigated to understand the distribution of the species in Colombia and other countries.

Because of its wide tolerance to relative humidity and temperature, *A*. *mixtum’s* geographical distribution extends from South Texas to the Pacific coast of Ecuador [30]. Moreover, the spatial distribution of *A*. *mixtum* was modeled from the records of presence-absence in the Neotropics and projected the presence of this tick in areas with high-temperature variability (including the Colombian Llanos Orientales), preferring sustained high temperatures, within a narrow range of average annual temperatures (~19-33°C) [51]. The Madrense semi-desert, which covers Southern Texas and a substantial part of northern Mexico, with an average annual temperature of ~23°C and an annual precipitation of only 112 mm in the town of Brownsville, Texas, (available from LandScope America: http://www.landscope.org/explore/natural_geographies/divisions/madrean_semidesert/) was projected as not suitable habitat for the establishment of *A*. *mixtum* by [51].

The other key variable for considering a suitable habitat for *A*. *mixtum* is the relative humidity (RH). It was showed that no *A*. *cajennense* s.l. female (renowned *A*. *mixtum*) survived more than a month in very dry environments (35% of RH) [87]. In our study region, we found large fluctuations in relative humidity. In 29 years, the extreme minimum RH value recorded was 61% for the Yopal region and, while for seven years it was as low as 30% at the Matepantano farm (Tables 6 and 7, respectively). Values below 40% relative humidity are uncommon or prolonged over time, and there are no data on their effect on *A*. *mixtum’s* development, survival, and population size of. Prolonged exposure to 56% relative humidity stimulated quiescence in some of this tick’s stages [49]. The death of all adults was recorded at 35% relative humidity and 33°C at the end of 30 days [87] (S5 Table).

In our study in the Matepantano farm, we found that the relative humidity fluctuates strongly within a few hours in a single day measure, as well as between seasons. In these habitats, leaf litter is often a shelter for ticks in extreme weather conditions, particularly because its structure retains moisture and provides a stable temperature for ticks [17,89]. In addition, the leaf litter may offer tick sub-adult ground hosts potential protection [90,91]. Higher relative humidity at ground level (leaf litter) may be associated with increased foliage density and decreased sunlight penetration, which could be related to *A*. *mixtum’s* preference for areas of dense vegetation compared to open areas with short grasses [92,93]. We hypothesize that larval and nymph stages might be more tolerant at low relative humidity and high temperature in the studied dry season (niche width).

In hydrophilic species of ixodids, such as *Ixodes uriae* or *Dermacentor variabilis*, sub-adult stages (larvae and nymphs) tend to be more prone to water stress from drying out than adults [94,95]. However, *A*. *mixtum* is considered one of the most dehydration-tolerant ticks of ixodids [49], which would indicate its xerophilic adaptation. Therefore, its tolerance to high temperatures and low relative humidity would explain its high population abundance in the studied dry season in the Matepantano farm. The mechanisms explaining how each stage of *A*. *mixtum* conserves body water in each habitat between seasons should be investigated.

Our seasonal abundance heterogeineity of immatures is consistent with previous findings [96], where the pre-adult stages of *A*. *sculptum* in Pantanal (Brazil) were found in the dry season but not in the wet season. According to these authors, the absence of immatures in the wet season in Pantanal can be explained by a seasonal pattern. [45,53] observed the synchronization of the development (behavioral diapause) of a stage of the *A*. *cajennense* s.l. The following stage will take place under favorable weather conditions according to the season. The immature-adult ratios we observed were relatively similar to those presented by [45,53], depending on the time of year.

We propose two hypotheses to explain the presence of the three post-embryonic stages of *A*. *mixtum* in both the dry and wet seasons and its relative abundance peak in the dry season in the Matepantano farm. The first is that there is only one generation per year because of a quiescent or diapause response of immature stages when experiencing extreme conditions, like prolonged flooding or high relative humidity during the rainy season, that comes out the following dry season. The second is that there are overlapping generations (several cohorts per year), even if the few surviving females generated a reproductive peak at the beginning of the dry season, following a population depression in the wet season because of the vulnerable immature stage’s death. We also believe that individuals from *A*. *mixtum* may have the ability to select a habitat outside the host, as is the case with other tick species [94,97]. Therefore, we recommend investigating this species’ habitat selection mechanisms, including its tolerance limits to environmental variables related to extreme events. Also, the factors involved in the detachment of the engorged tick from the host when it perceives a habitat that is favorable should be studied, a process that we consider not to be random.

The seasonal flooding of soils in some regions of Brazil could be unfavorable to *A*. *sculptum* ticks, which prefer drained soils in open areas with shrubs and riparian forests [98]. In the laboratory, engorged females from *Amblyomma auricularium* were resistant to water stress when placed under immersion in distilled water for up to 96 hours [99]. However, the reduced progeny and the delayed oviposition time were attributed to diapause. It has also been shown that the eggs of *A*. *americanum* withstand underwater immersion periods of up to a week, without altering incubation time or hatching success [100]. Anecdotally, we observed that a few larvae of *A*. *mixtum* could survive up to one week under complete immersion in distilled water under laboratory conditions (~26°C, 70–80% RH, within an incubator) at the Special Bacteriology Laboratory of the Pontificia Universidad Javeriana (Bogotá, D.C., Colombia).

In this regard, the King Grass Crop habitat is temporary, composed of non-perennial grass *P*. *hybridum*, and its use and preference by *A*. *mixtum* are opportunistic. Our observations coincide with the hypothesis of physiological plasticity indicated for the *A*. *cajennense* s.l. [101], where a set of habitat variables can explain these ticks’ survival, not by strict host specificity. By contrast, ticks avoided the Riparian Forest habitat according to the calculated use and preference rates; this could be related to different factors (e.g., a greater number of natural enemies, the constant export of ticks by wild animals to neighboring habitats, and some abiotic factors that exceed the ticks’ tolerance).

For its part, the fundamental niche of *A*. *mixtum* is determined by the tick’s tolerance range for the conditions it faces (i.e., temperature, humidity, solar irradiance), variables that define the breadth of habitat it colonizes. Thus, the niche width of *A*. *mixtum* is the range of habitats, with their range of resources, in which the growth rate of their population is positive. Briefly, a generalist species is actually composed of different specialist individuals, called individual specialization, to increase their **fitness** as an adaptive strategy in unpredictable environments [102] (e.g., *A*. *mixtum* larvae had the highest niche width in the dry season, while adults had it on the wet season in our study). It makes sense for a tick species to have a broad niche amplitude since the longest period of its life cycle is outside the host [101] and can occur in different types of habitats. Conversely, a tick species specialized in one habitat type may be disadvantaged by habitat loss, fragmentation, or transformation [103]. According to the above, the fragmentation of habitats in an agroecosystem like the Matepantano farm impacts the availability and diversity of habitats and hosts, thus the abundance of ticks at different times of the year, where the areas, cover, and types of vegetation change. This fragmentation of the landscape [103,104] could be beneficial for *A*. *mixtum*, offering a wide variety of environmental possibilities that favor their survival, reproduction, and niche breadth.

The significance of the *A*. *mixtum* free-living stages niche amplitude should not be extrapolated beyond the data supported, which is a high ability to colonize diverse environments. However, different biotic relationships along with tick survival behaviors might play a key role in explaining seasonal abundance and should be further studied in the region. We hypothesize that a continuous and diverse supply of *A*. *mixtum* hosts in the Matepantano farm would increase the connectivity between different habitats and expand the generalist species’ capacity to tolerate different conditions and have a wide range of resources to maintain the population over time. The niche width of *A*. *mixtum* in the Matepantano farm was higher at both times of the year for adults. This result may indicate the adult stage’s ability to resist environmental changes and use different resources in changing or altered habitats, probably favoring its spatial dispersion because of its wide range of hosts [30,34,35,48].

If the wet season drastically affects the *A*. *mixtum* population size in the following year, how can be explained the persistence of *A*. *mixtum* in the Matepantano farm? To answer this hypothesis, we used a mathematical inference. We calculated the intrinsic rate of population growth = $R(n_{t-1}/n_{t})$, which relates the population abundance (n) in a time (t+1) with respect to a previous time (t); being R = 1 a stable population, >1 growing and <1 in decline. Thus, if we divide *A*. *mixtum’s* total abundance values quantified in the wet season (266) by those quantified in the dry season (6,733), the result is an R = 0.039, suggesting population decrease between both the dry and wet seasons for the same year. That is, a population loss rate of 96.1% (1–0.039; or 100% - 3.9%).

We quantified 77 females in the wet season, however, not all of them will manage to reach engorgement during this season. By probability, many eggs will not survive or develop into larvae. Furthermore, assuming that the effective number of larvae in the following dry season is equal to the previous season, the R value between the females accounted for in the wet season and the expected larvae in the dry season of the following year it would be 2,533 /77 = 32.89 → R = (1–32.89) = 31.89, yielding a population growth of 3,189% (31.89*100%). To know the net balance in the population growth between the dry season of the quantified year and the dry season of the following year, we multiply the values of R, so 0.039 _X_ 32.89 = 1.28 (R = 1–1.28 = 0.28 or 0.28 x 100% = 28%), resulting in a net population gain of 28%. This calculation suggests that, even with massive loss of individuals in the wet season (one order of magnitude), the population would be increased by 28% the following year, explaining the testimonies by the inhabitants of the Matepantano farm on the persistence of the species over time. This population persistence could be explained because a low number of engorged females are enough to produce thousands of eggs and subsequent larvae to maintain a positive population size. Another explanation could be a continuous supply of individuals (rescue effect) from neighboring habitats, by hosts, part of the so-called source-sink population dynamics [6].

Considering the potential hosts seen by people in the Matepantano farm, the functional connectivity of the fragmented agroecosystem landscape, particularly between habitats of King Grass Crop with Cocoa Crop and Riparian Forest may be hypothetically occurring through small mammals (marsupials and armadillos) and capybaras (*Hydrochoerus hydrochaeris*) (Fig 8). Some hosts functionally connect habitats, either because these hosts find food in these habitats [18,105] or refuge from potential predators [17]. As opportunistic species on modified fragmented and crop-based systems, ticks take advantage of this dispersal mechanism even when the primary hosts are absent [22] or have been anthropogenically restricted [106], seeing a possibility of habitat expansion [107].

Although there is no data on the horizontal movement of each *A*. *mixtum* stage, field experiments in Texas [49] indicated that in the vertical displacement in search of hosts, a third of the nymphs exceeded 30 cm from the ground and 3% of the adults were found between 10 and 60 cm, with a maximum of 115 cm, favoring their hunting strategy over their dispersion and feeding. Less vertical movement has been reported in the related species *A*. *sculptum* in Pantanal (Brazil) [96] with 15 to 50 cm of vertical climbing. We need to investigate the abilities of *A*. *mixtum* in its different stages to move between habitats.

Our findings validate modeling results for *A*. *mixtum* in the Neotropics [51] and in Colombia [108], which point out different types of vegetation cover, as well as hot and dry seasons, as favorable for the presence of this tick. Because of its high thermal tolerance and niche amplitude, *A*. *mixtum* colonizes several types of habitats, providing diverse resources and climatic conditions in the Matepantano farm. Certainly, if coupled with functional connectivity by hosts, this would allow *A*. *mixtum* to adapt and survive in changing habitats. These characteristics of this tick represents a serious challenge for the human and veterinary public health systems.

We consider, based on the results of our study, that *A*. *mixtum* is more vulnerable in the wet season, a rainy time characterized by floodings in the region. Reducing the risk of rickettsial infection in humans should be the goal of control measures on the Matepantano farm and in the Llanos Orientales region. It is recommended to monitor the populations of *A*. *mixtum* free-living stages in the different habitats of the Matepantano farm, as well as their parasitic populations on domestic and wild animals, in relation to the climate and microclimate at different times of the year. Such information will be useful in determining the local distribution of *A*. *mixtum* and to identify those key factors that explain the tolerance of the tick to extreme values of temperature and RH to plan effective and specific strategies of control of its off-host stages in the region.

## Conclusion

*A*. *mixtum* presented preference for diverse environments in the Matepantano farm, as well as niche amplitude and tolerance to strong environmental fluctuations. All stages of *A*. *mixtum* use the Riparian Forest but do not prefer it; it prefers the three changing and transformed habitats around the Riparian Forest. The population was more abundant in the dry season (larvae and nymphs) and decreased by one order of magnitude in the wet season (more adults, relatively). In the wet season, the immature stages were less abundant and control measures might be more effective if they are focused during this time of the year.

Further research should be developed to understand the morphophysiological or behavioral strategies and adaptations of *A*. *mixtum* to tolerate wide fluctuations and extreme changes in climate and microclimate. Several variables that falling within the scope of ‘functional habitat’ (e. g., host richness within each habitat associated to *A*. *mixtum*; host parasitic load; host biotic interactions with natural enemies; and host role in landscape connectivity, maintenance, and persistence of tick populations) should be included in future research. Also, tick intrinsic variables, like photoperiod behavior and development, might help explain the parasitic and non-parasitic population peaks of all *A*. *mixtum* stages in both dry and wet seasons to determine generational overlap, and to distinguish strategies for survival for every developmental stage. The population control of *A*. *mixtum* should be carried out at the time of greatest vulnerability (e. g., the studied wet season), where the population size is low.

## Supporting information

S1 Fig Morphological details of *A*. *mixtum* female specimens collected from one CO2 trap (Y-T031) in the Riparian Forest habitat in the wet season (August/2019). (PDF)

S2 Fig Morphological details of *A*. *mixtum* male specimens collected from one CO2 trap (Y-T031) in the Riparian Forest habitat in the wet season (August/2019). (PDF)

S1 Table Codification for every ice trap (N = 24) with effective tick collection in the dry season, including sample code, habitat and GPS coordinates. (DOCX)

S2 Table Codification for every transect (N = 22) with effective tick collection in the dry season, including sample code, habitat and GPS coordinates. (DOCX)

S3 Table Codification for every ice trap (N = 31) with effective tick collection in the wet season, including sample code, habitat and GPS coordinates. (DOCX)

S4 Table Codification for every transect (N = 22) with effective tick collection in the wet season, including sample code, habitat and GPS coordinates. (DOCX)

S5 Table Abiotic variables recorded for *Amblyomma mixtum* in literature. (DOCX)

S1 Appendix Procedure used for drone orthomosaic mapping. (DOCX)

S2 Appendix Procedure for vegetation cover estimation within each habitat. (DOCX)

S3 Appendix Description of the making process for the ice traps and for the white flannelettes to collect free-living ticks on the field. (DOCX)

S4 Appendix Testing of 21 larva, nymph, and adult *Amblyomma mixtum* DNA samples (individual specimens or pools) for two mitochondrial genes and one nuclear gene. (DOCX)
