# Supplementary material for: Amblyomma mixtum free-living stages: Inferences on dry and wet seasons use, preference, and niche width in an agroecosystem (Yopal, Casanare, Colombia)
Source: PLoS One. 2022 Apr 6;17(4):e0245109. doi: 10.1371/journal.pone.0245109 (PMC8986011; doi:10.1371/journal.pone.0245109)
Supplement: S1 Fig — (PDF) [file pone.0245109.s001.pdf]

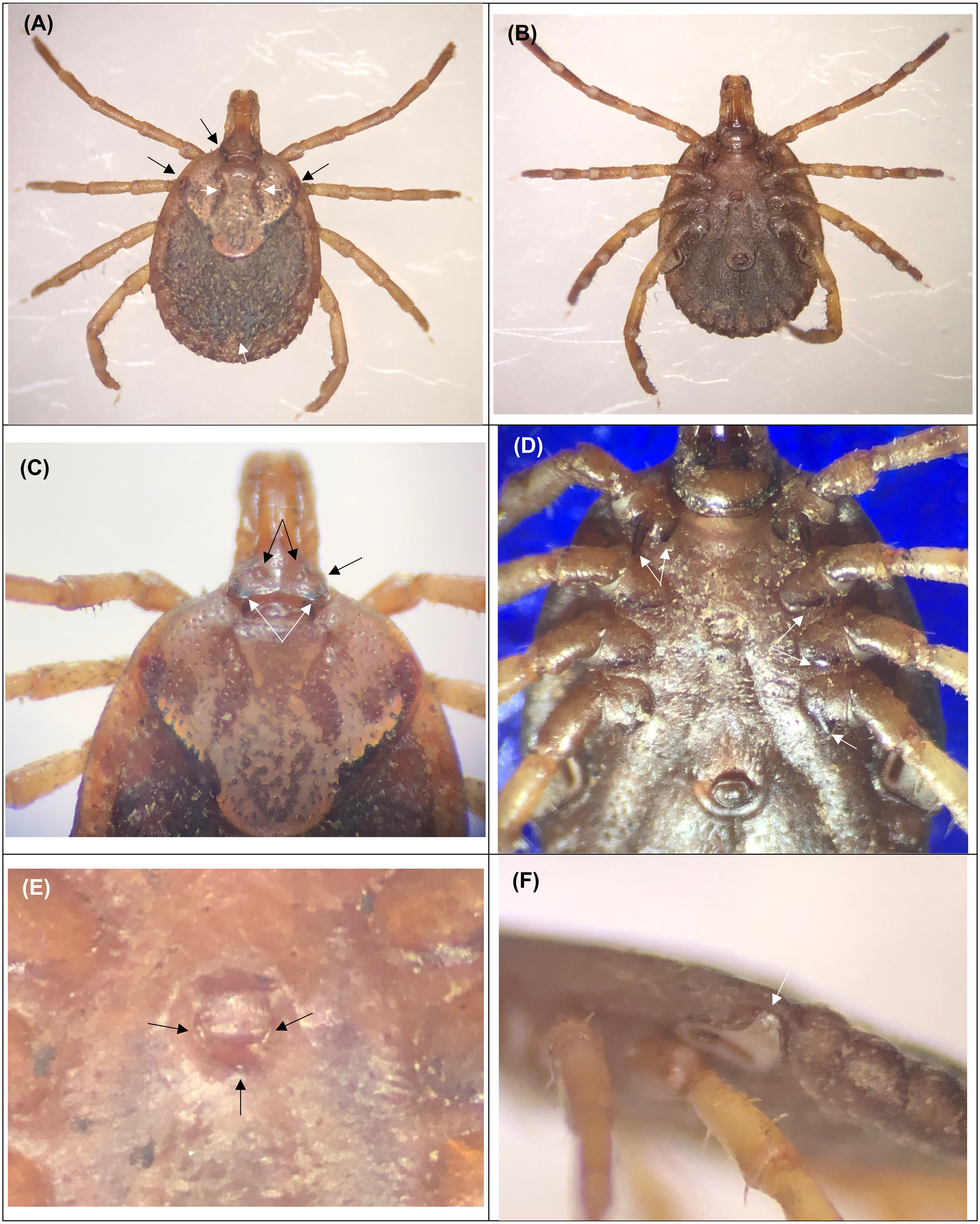

S1 Fig. Morphological details of *A. mixtum* female specimens collected from one CO2 trap (Y-T031) in the Riparian Forest habitat in the wet season (August/2019). Two female specimens were used: F1 and F2. **(**A) Female (F1), dorsal view where black arrows indicate scapulae pointed and position of the eyes in the scutum, and white arrows show the cervical grooves on the scutum with a deep and obliquely linear characteristic (from lateral to medial) and a marginal groove complete delimiting all festoons. (B) Female (F1), ventral view. (C) Female (F1), dorsal view of basis capituli, where

black arrows indicate the sub-rectangular characteristic of the basis capituli and cornua rounded, while white arrows show the porose areas being oval and diverging anteriorly. (D) Female (F1), ventral view showing coxae I-IV; white arrows indicate coxae I with 2 distinct spurs being the external longer than the internal one, coxae II-III having an ridge-like edge, and coxae IV having a distinct, short, and rounded internal spur. (E) Female (F3), ventral view of the genital aperture; black arrows point out the U-shaped form of the genital aperture and the presence of 2 narrow lateral flaps. (F) Female (F2), lateral left view showing the spiracular plates and their comma-shaped characteristic with a small causal process (white arrow) as wide as the adjacent festoon
