# Supplementary material for: Amblyomma mixtum free-living stages: Inferences on dry and wet seasons use, preference, and niche width in an agroecosystem (Yopal, Casanare, Colombia)
Source: PLoS One. 2022 Apr 6;17(4):e0245109. doi: 10.1371/journal.pone.0245109 (PMC8986011; doi:10.1371/journal.pone.0245109)
Supplement: S2 Fig — (PDF) [file pone.0245109.s002.pdf]

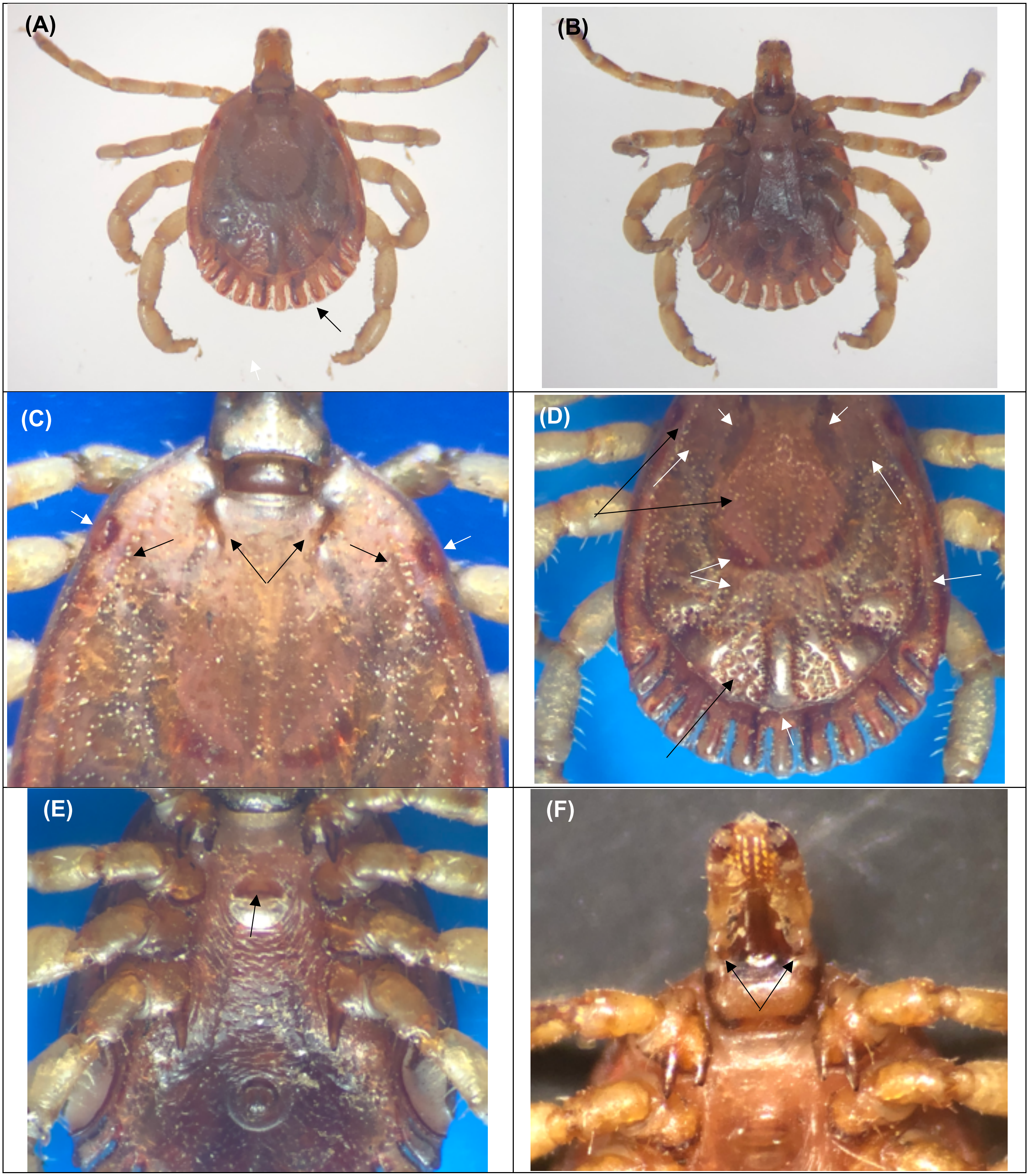

S2 Fig. Morphological details of *A. mixtum* male specimens collected from one CO2 trap (Y-T031) in the Riparian Forest habitat in the wet season (August/2019). One male specimen was used (M1). **(**A) Dorsal view where black arrows the body outline round. (B) Ventral view. (C) Anterior part of the scutum; black arrows show the cervical grooves deep, short, comma-shaped, as well as a line of deep punctations reaching level of eyes; white arrows indicate the position of the flat eyes at the antero-lateral edges of the scutum. (D) Posterior part of the scutum; white arrows indicate a deep marginal groove complete, which is delimitating all festoons up to level of coxa IV, but it is less deeper when reaching the level of eyes; also, white arrows show the characteristic pattern of the spots in the scutum (cervical spots large and elongated posteriorly; antero-accessory spots large and distinct; and branches of limiting spots broad, posterior branches of limiting spots not fused posteriorly); black arrows indicate the posterior scutum moderately punctated with large and shallow punctations deeper, while the anterior part of scutum have finer and less numerous punctations, but deeper in marginal areas. (E) Ventral view of the central area of the body showing coxae I-IV; the black arrow indicate the U-shaped form of the genital aperture at level of coxae II. (F) Capitulum, ventral view; the black arrow point out the small, blunt spur that projects posteriorly in palp segment I.
