# Supplementary material for: Amblyomma mixtum free-living stages: Inferences on dry and wet seasons use, preference, and niche width in an agroecosystem (Yopal, Casanare, Colombia)
Source: PLoS One. 2022 Apr 6;17(4):e0245109. doi: 10.1371/journal.pone.0245109 (PMC8986011; doi:10.1371/journal.pone.0245109)
Supplement: S1 Table — (DOCX) [file pone.0245109.s003.docx]

**S1 Table. Codification for every ice trap (N = 24) with effective tick collection in the dry season, including sample code, habitat and GPS coordinates.**

| **Trap No.** | **Sample ID** | **Date** | **Habitat** | **GPS Label** | **Latitude** | **Longitude** |
| --- | --- | --- | --- | --- | --- | --- |
| T01 | Y-T001 | 9-Feb-19 | Star Grass Paddock | 163-T01 | 5.323408 | -72.288978 |
| T02 | Y-T002 | 9-Feb-19 | Star Grass Paddock | 163-T02 | 5.323874 | -72.289073 |
| T03 | Y-T003 | 9-Feb-19 | Star Grass Paddock | 163-T03 | 5.324001 | -72.289284 |
| T04 | Y-T004 | 9-Feb-19 | Star Grass Paddock | 163-T04 | 5.324041 | -72.289339 |
| T05 | Y-T026, Y-T029 | 9-Feb-19 | Star Grass Paddock | ND | ND | ND |
| T07 | Y-T015 | 10-Feb-19 | Star Grass Paddock | 162-T07 | 5.323624 | -72.288918 |
| T08 | Y-T016 | 10-Feb-19 | Star Grass Paddock | 162-T08 | 5.323005 | -72.288963 |
| T16 | Y-T018 | 10-Feb-19 | King Grass Crop | 165-T16 | 5.323610 | -72.289125 |
| T17 | Y-T019 | 10-Feb-19 | King Grass Crop | 165-T17 | 5.323617 | -72.289212 |
| T18 | Y-T020 (A, B, C) | 10-Feb-19 | King Grass Crop | 165-T18 | 5.323587 | -72.289296 |
| T19 | Y-T021 | 10-Feb-19 | King Grass Crop | 165-T19 | 5.323681 | -72.289308 |
| T20 | Y-T023 | 10-Feb-19 | King Grass Crop | 165-T20 | 5.323574 | -72.289359 |
| T06 | Y-T012 | 10-Feb-19 | Riparian Forest | 162-T06 | 5.323953 | -72.288882 |
| T09 | Y-T017 | 10-Feb-19 | Riparian Forest | 162-T09 | 5.322958 | -72.288892 |
| T10 | Y-T022, Y-T010-B | 10-Feb-19 | Riparian Forest | 162-T10 | 5.323005 | -72.288929 |
| T21 | Y-T006 | 10-Feb-19 | Riparian Forest | ND | ND | ND |
| T22 | Y-T007 | 10-Feb-19 | Riparian Forest | ND | ND | ND |
| T23 | Y-T008 | 10-Feb-19 | Riparian Forest | ND | ND | ND |
| T23 | Y-T009 | 10-Feb-19 | Riparian Forest | ND | ND | ND |
| T24 | Y-T010 | 10-Feb-19 | Riparian Forest | ND | ND | ND |
| T24 | Y-T024 | 10-Feb-19 | Riparian Forest | ND | ND | ND |
| T25 | Y-T011 | 10-Feb-19 | Riparian Forest | ND | ND | ND |
| T12 | Y-T025, Y-T014 | 10-Feb-19 | Cocoa Crop | 164-T12 | 5.324104 | -72.288755 |
| T13 | Y-T027 | 10-Feb-19 | Cocoa Crop | 164-T13 | 5.324324 | -72.288508 |
| T14 | Y-T013 | 10-Feb-19 | Cocoa Crop | 164-T14 | 5.324517 | -72.288928 |
| T15 | Y-T028 | 11-Feb-19 | Cocoa Crop | 164-T15 | 5.324406 | -72.289261 |

ND = no data; dry ice traps were placed at those sites and samples were collected, but GPS coordinates were not recorded.
