# Supplementary material for: Amblyomma mixtum free-living stages: Inferences on dry and wet seasons use, preference, and niche width in an agroecosystem (Yopal, Casanare, Colombia)
Source: PLoS One. 2022 Apr 6;17(4):e0245109. doi: 10.1371/journal.pone.0245109 (PMC8986011; doi:10.1371/journal.pone.0245109)
Supplement: S2 Table — (DOCX) [file pone.0245109.s004.docx]

**S2 Table. Codification for every transect (N = 22) with effective tick collection in the dry season, including sample code, habitat and GPS coordinates.**

| **Transect No.** | **Sample ID** | **Date** | **Habitat** | **GPS Label** | **Latitude** | **Longitude** |
| --- | --- | --- | --- | --- | --- | --- |
| S14 | Y-S014 | 11-Feb-19 | Star Grass Paddock | 163 | 5.322637 | -72.289037 |
| S15 | Y-S015 | 11-Feb-19 | Star Grass Paddock | 163 | 5.322637 | -72.289037 |
| S16 | Y-S016 | 11-Feb-19 | Star Grass Paddock | 164 | 5.323163 | -72.289095 |
| S17 | Y-S017 | 11-Feb-19 | Star Grass Paddock | 164 | 5.323163 | -72.289095 |
| S18 | Y-S018 | 11-Feb-19 | Star Grass Paddock | 164 | 5.323163 | -72.289095 |
| S19 | Y-S019 | 11-Feb-19 | Star Grass Paddock | 166 | 5.315863 | -72.301650 |
| S20 | Y-S020 | 11-Feb-19 | Star Grass Paddock | 166 | 5.315863 | -72.301650 |
| S09 | Y-S002 | 8-Feb-19 | King Grass Crop | ND | ND | ND |
| S10 | Y-S005 | 9-Feb-19 | King Grass Crop | ND | ND | ND |
| S11 | Y-S006 | 9-Feb-19 | King Grass Crop | ND | 5.324015 | -72.290033 |
| S12 | Y-S007 | 9-Feb-19 | King Grass Crop | ND | 5.324015 | -72.290033 |
| S13 | Y-S008 | 9-Feb-19 | King Grass Crop | ND | 5.324015 | -72.290033 |
| S23 | Y-S009 | 9-Feb-19 | King Grass Crop | ND | 5.324017 | -72.290035 |
| S07 | Y-S010 | 10-Feb-19 | Riparian Forest | 160 | 5.322239 | -72.286958 |
| S08 | Y-S011 | 10-Feb-19 | Riparian Forest | 161 | 5.322927 | -72.288894 |
| S21 | Y-S021 | 11-Feb-19 | Riparian Forest | 167 | 5.315299 | -72.301155 |
| S22 | Y-S022 | 11-Feb-19 | Riparian Forest | 167 | 5.315299 | -72.301155 |
| S01 | Y-S001 | 8-Feb-19 | Cocoa Crop | 149 | 5.323415 | -72.286978 |
| S03 | Y-S003 | 8-Feb-19 | Cocoa Crop | 152 | 5.323540 | -72.287159 |
| S04 | Y-S004 | 8-Feb-19 | Cocoa Crop | 154 | 5.323193 | -72.287224 |
| S05 | Y-S012 | 10-Feb-19 | Cocoa Crop | 164-T14 | 5.324517 | -72.288928 |
| S06 | Y-S013 | 10-Feb-19 | Cocoa Crop | 164-T14 | 5.324517 | -72.288928 |

ND = no data; transects were carried out those sites and samples were collected, but GPS coordinates were not recorded in some cases.
