# Supplementary material for: Amblyomma mixtum free-living stages: Inferences on dry and wet seasons use, preference, and niche width in an agroecosystem (Yopal, Casanare, Colombia)
Source: PLoS One. 2022 Apr 6;17(4):e0245109. doi: 10.1371/journal.pone.0245109 (PMC8986011; doi:10.1371/journal.pone.0245109)
Supplement: S3 Table — (DOCX) [file pone.0245109.s005.docx]

**S3 Table. Codification for every ice trap (N = 31) with effective tick collection in the wet season, including sample code, habitat and GPS coordinates.**

| **Trap No.** | **Sample ID** | **Date** | **Habitat** | **GPS Label** | **Latitude** | **Longitude** |
| --- | --- | --- | --- | --- | --- | --- |
| T18 | … | 16-Aug-19 | Star Grass Paddock | 183 T18 P Bajo | 5.32043 | -72.29191 |
| T19 | … | 16-Aug-19 | Star Grass Paddock | 183 T19 P Bajo | 5.32005 | -72.29179 |
| T20 | … | 16-Aug-19 | Star Grass Paddock | 183 T20 P Bajo | 5.31992 | -72.29152 |
| T21 | … | 16-Aug-19 | Star Grass Paddock | 183 T21 P Bajo | 5.31977 | -72.29108 |
| T22 | … | 16-Aug-19 | Star Grass Paddock | 183 T22 P Bajo | 5.32000 | -72.29087 |
| T23 | … | 16-Aug-19 | Star Grass Paddock | 183 T23 P Bajo | 5.32015 | -72.29117 |
| T24 | Y-T047 | 17-Aug-19 | Star Grass Paddock | ND | ND | ND |
| T25 | Y-T048 | 17-Aug-19 | Star Grass Paddock | ND | ND | ND |
| T2 | Y-T032 | 16-Aug-19 | King Grass Crop | 183 T2 P Alto | 5.32381 | -72.28905 |
| T3 | Y-T033 | 16-Aug-19 | King Grass Crop | 183 T3 P Alto | 5.32395 | -72.28925 |
| T4 | Y-T034 | 16-Aug-19 | King Grass Crop | 183 T4 P Alto | 5.32391 | -72.28929 |
| T8 | Y-T038 | 16-Aug-19 | King Grass Crop | 183 T8 P Alto | 5.32350 | -72.28934 |
| T9 | Y-T039 | 16-Aug-19 | King Grass Crop | 183 T9 P Alto | 5.32351 | -72.28947 |
| T10 | Y-T040 | 16-Aug-19 | King Grass Crop | 183 T10 P Alto | 5.32336 | -72.28956 |
| T1 | Y-T031 | 16-Aug-19 | Riparian Forest | 183 T1 Bosque | 5.32367 | -72.28893 |
| T5 | Y-T035 | 16-Aug-19 | Riparian Forest | 183 T5 Bosque | 5.32325 | -72.28896 |
| T6 | Y-T036 | 16-Aug-19 | Riparian Forest | 183 T6 Bosque | 5.32294 | -72.28888 |
| T7 | Y-T037 | 16-Aug-19 | Riparian Forest | 183 T7 Bosque | 5.32389 | -72.28897 |
| T16 | Y-T046 | 16-Aug-19 | Riparian Forest | 183 T16 Bosque | 5.32382 | -72.28870 |
| T26 | Y-T049 | 17-Aug-19 | Riparian Forest | ND | ND | ND |
| T27 | Y-T050 | 17-Aug-19 | Riparian Forest | ND | ND | ND |
| T28 | Y-T051 | 17-Aug-19 | Riparian Forest | ND | ND | ND |
| T29 | Y-T052 | 17-Aug-19 | Riparian Forest | ND | ND | ND |
| T30 | Y-T053 | 17-Aug-19 | Riparian Forest | ND | ND | ND |
| T31 | Y-T054 | 17-Aug-19 | Riparian Forest | ND | ND | ND |
| T17 | … | 16-Aug-19 | Riparian Forest | 183 T17 Bosque | 5.32393 | -72.28915 |
| T11 | Y-T041 | 16-Aug-19 | Cocoa Crop | 183 T11 Cacao | 5.32422 | -72.28874 |
| T12 | Y-T042 | 16-Aug-19 | Cocoa Crop | 183 T12 Cacao | 5.32453 | -72.28923 |
| T13 | Y-T043 | 16-Aug-19 | Cocoa Crop | 183 T13 Cacao | 5.32444 | -72.28878 |
| T14 | Y-T044 | 16-Aug-19 | Cocoa Crop | 183 T14 Cacao | 5.32351 | -72.28827 |
| T15 | Y-T045 | 16-Aug-19 | Cocoa Crop | 183 T15 Cacao | 5.32317 | -72.28783 |

ND = no data; dry ice traps were placed at those sites and samples were collected, but GPS coordinates were not recorded.

(…) = sample not collected because ticks were not found.
