# Supplementary material for: Amblyomma mixtum free-living stages: Inferences on dry and wet seasons use, preference, and niche width in an agroecosystem (Yopal, Casanare, Colombia)
Source: PLoS One. 2022 Apr 6;17(4):e0245109. doi: 10.1371/journal.pone.0245109 (PMC8986011; doi:10.1371/journal.pone.0245109)
Supplement: S4 Table — (DOCX) [file pone.0245109.s006.docx]

**S4 Table. Codification for every transect (N = 22) with effective tick collection in the wet season, including sample code, habitat and GPS coordinates.**

| **Transect No.** | **Sample ID** | **Date** | **Habitat** | **GPS Label** | **Latitude** | **Longitude** |
| --- | --- | --- | --- | --- | --- | --- |
| S22 | Y-S038 | 17-Aug-19 | Star Grass Paddock | 183 S22A P Baj | 5.32069 | -72.29198 |
| S23 | Y-S039 | 17-Aug-19 | Star Grass Paddock | 183 S23A P Baj | 5.32030 | -72.29189 |
| S24 | … | 17-Aug-19 | Star Grass Paddock | 183 S24A P Baj | 5.31999 | -72.29183 |
| S25 | … | 17-Aug-19 | Star Grass Paddock | 183 S25A P Baj | 5.31981 | -72.29130 |
| S26 | … | 17-Aug-19 | Star Grass Paddock | 183 S26A P Baj | 5.31998 | -72.29078 |
| S1 | Y-S031 | 17-Aug-19 | King Grass Crop | 183 S1A P Alto | 5.32365 | -72.28910 |
| S20 | Y-S036 | 17-Aug-19 | King Grass Crop | ND | ND | ND |
| S2 | … | 17-Aug-19 | King Grass Crop | 183 S2A P Alto | 5.32385 | -72.28925 |
| S3 | … | 17-Aug-19 | King Grass Crop | 183 S3A P Alto | 5.32394 | -72.28933 |
| S4 | … | 17-Aug-19 | King Grass Crop | 183 S4A P Alto | 5.32377 | -72.28921 |
| S5 | … | 17-Aug-19 | King Grass Crop | 183 S5A P Alto | 5.32357 | -72.28919 |
| S6 | … | 17-Aug-19 | King Grass Crop | 183 S6A P Alto | 5.32365 | -72.28929 |
| S15 | … | 17-Aug-19 | Riparian Forest | 183 15A Bosqe | 5.32379 | -72.28886 |
| S16 | Y-S034 | 17-Aug-19 | Riparian Forest | 183 S16A Bosqe | 5.32403 | -72.28905 |
| S17 | Y-S035 | 17-Aug-19 | Riparian Forest | 183 S17A Bosqe | 5.32311 | -72.28907 |
| S21 | Y-S037 | 17-Aug-19 | Riparian Forest | 183 S21A Bosqe | 5.32296 | -72.28890 |
| S7 | … | 17-Aug-19 | Cocoa Crop | 183 S7A Cacao | 5.32394 | -72.28861 |
| S8 | … | 17-Aug-19 | Cocoa Crop | 183 S8A Cacao | 5.32444 | -72.28877 |
| S9 | … | 17-Aug-19 | Cocoa Crop | 183 S9A Cacao | 5.32395 | -72.28843 |
| S14 | … | 17-Aug-19 | Cocoa Crop | 183 S14A Cacao | 5.32336 | -72.28809 |
| S11 | Y-S032 | 17-Aug-19 | Cocoa Crop | 183 S11A Cacao | 5.32407 | -72.28848 |
| S12 | Y-S033 | 17-Aug-19 | Cocoa Crop | ND | ND | ND |

ND = no data; transects were carried out at those sites and samples were collected, but GPS coordinates were not recorded.

(…) = sample not collected because ticks were not found.
