# Supplementary material for: Amblyomma mixtum free-living stages: Inferences on dry and wet seasons use, preference, and niche width in an agroecosystem (Yopal, Casanare, Colombia)
Source: PLoS One. 2022 Apr 6;17(4):e0245109. doi: 10.1371/journal.pone.0245109 (PMC8986011; doi:10.1371/journal.pone.0245109)
Supplement: S5 Table — (DOCX) [file pone.0245109.s007.docx]

**S5 Table. Abiotic variables recorded for *Amblyomma mixtum* in literature.**

| **Stage** | **Place** | **Variable** | **Mean** | **Range** | **Observations** | **Ref.** |
| --- | --- | --- | --- | --- | --- | --- |
| Female | Laboratory | Constant  Temperature | … | 23-33ºC | 0% of mean survival at 23ºC, 85% (RH) and 2,98 mmHg (SDe) in 682.5 days (range: 625-770 days).  0% of mean survival at 33ºC, 35% (RH) and 42,47 mmHg (SDe) in 22.3 days (range: 20-30 days). | [1] |
|  |  | Constant relative humidity (RH) | … | 35-85% |  |  |
| Male |  |  |  |  | 0% of mean survival at 23ºC, 85% (RH) and 2,98 mmHg (SDe) 641.2 days (range: 641,2 days).  0% of mean survival at 33ºC, 35% (RH) and 42,47 mmHg (SDe) in 28.5 days (range: 27-30 days). |  |
|  |  | Saturation deficit (SDe) | … | 2,98-24,47 mmHg |  |  |
| Nymphs | Acrylic actographic chamber in the field (Texas) | Constant relative humidity (RH) | … | 56-95% | Nymphs kept at 95% RH started questing earlier than those at 56%.  Nymphs kept over long periods at 56% RH became quiescent before than those at 95% RH. | [2] |
| Adults |  |  |  |  | More adults quested to greater heights at 95%RH than those at 56%  At lower RH, ticks were still viable but quiescent. |  |
| Adults, nymphs | Panama (national wide sampling) | Mean temperature | … | 20,9-32,4ºC* | The current distribution of *A. mixtum* in Panama seems to be limited to areas below 800 m.a.s.l., particularly bush areas on the Pacific slope and less frequent on fields with introduced grass crops. Established populations of *A. mixtum* were not found above 1,200 m.a.s.l. in areas with a combination of a low mean temperature (<15ºC) and a high RH (>85%). | [3] |
|  |  | Annual mean precipitation | 2214,3 mm | 3732-1111 mm |  |  |
| Adults, nymphs | Tamaulipas (Mexico) | Annual Temperature | 27,5ºC | 23,3-33ºC | Data of environmental conditions where tick infested cattle was found. | [4] |
|  |  | Annual relative humidity | 76,2% | 63,6-95% |  |  |
|  |  | Annual mean precipitation | 139,7 | 4-420 |  |  |
| Adults, nymphs | Tabasco (Mexico) | Annual mean temperature | 27ºC | 18-36ºC | Adults and nymphs where collected from sheep throughout the year (including rainy and dray seasons) from Sep/2014 to Sep/2015. | [5] |
|  |  | Annual mean precipitation | 2.550 mm | … |  |  |

*Mean temperature of the coldest and the warmest month, respectively.

**REFERENCES**

1. Strey OF, Teel PD, Longnecker MT, Needham GR. Survival and Water-Balance Characteristics of Unfed Adult *Amblyomma cajennense* (Acari: Ixodidae). J Med Entomol. 1996;33: 63–73. doi:10.1093/jmedent/33.1.63

2. Beck DL, Orozco JP. Diurnal questing behavior of *Amblyomma mixtum* (Acari: Ixodidae). Exp Appl Acarol. 2015;66: 613–621. doi:10.1007/s10493-015-9928-x

3. Bermúdez SE, Castro AM, Trejos D, García GG, Gabster A, Miranda RJ, et al. Distribution of Spotted Fever Group Rickettsiae in Hard Ticks (Ixodida: Ixodidae) from Panamanian Urban and Rural Environments (2007–2013). Ecohealth. 2016;13: 274–284. doi:10.1007/s10393-016-1118-8

4. Almazán C, Torres-Torres A, Torres-Rodríguez L, Soberanes-Céspedes N, Ortiz-Estrada M. Aspectos biológicos de *Amblyomma mixtum* (Koch, 1844) en el noreste de México. Rev Quehacer Científico en Chiapas. 2016;11: 10–19. Available: https://www.dgip.unach.mx/images/pdf-REVISTA-QUEHACERCIENTIFICO/2016-jul-dic/Aspectos_biologicos_de_Amblyomma_mixtum_.pdf

5. Coronel-Benedett KC, Ojeda-Robertos NF, González-Garduño R, Ibañez FM, Rodríguez-Vivas RI. Prevalence, intensity and population dynamics of hard ticks (Acari: Ixodidae) on sheep in the humid tropics of Mexico. Exp Appl Acarol. 2018;74: 99–105. doi:10.1007/s10493-017-0195-x
