# Supplementary material for: Amblyomma mixtum free-living stages: Inferences on dry and wet seasons use, preference, and niche width in an agroecosystem (Yopal, Casanare, Colombia)
Source: PLoS One. 2022 Apr 6;17(4):e0245109. doi: 10.1371/journal.pone.0245109 (PMC8986011; doi:10.1371/journal.pone.0245109)
Supplement: S1 Appendix — (DOCX) [file pone.0245109.s008.docx]

**S1 Appendix. Procedure used for drone orthomosaic mapping according to the drone imaging service that was hired.**

- A digital camera **FC6310** (8.8 mm focal distance with 4864 x 3648 pixel resolution and 2.61 µm pixel size) was used to take a total of 561 aerial pictures from Matepantano farm and surroundings through three consecutive flight missions.
- All flight missions were carried out through the **DroneDeploy®** app, which generates polygons over the study areas and later they are identified on a free-access satelital map provide by Google. Lateral and frontal photo overlay was 70% and 85%, respectively.
- **Agisoft Photoscan®** software was used for processing all digital images taken, create an image orthomosaic and drawing over such orthomosaic recognizable habitat limits from the field (Figure 1).
- The ‘Editor’ tool from the **ArcGIS Desktop®** software (Esri, Redlands, CA, USA) was used to generate several polygons over the image orthomosaic in order to distinguish four types of dominant terrestrial vegetation or **habitats**: riparian forest, cacao crop, King Grass crop, and Star Grass paddocks (Figure 1).
- Several **topology corrections** were made to avoid gaps between polygons and overlay areas.
