# Supplementary material for: Amblyomma mixtum free-living stages: Inferences on dry and wet seasons use, preference, and niche width in an agroecosystem (Yopal, Casanare, Colombia)
Source: PLoS One. 2022 Apr 6;17(4):e0245109. doi: 10.1371/journal.pone.0245109 (PMC8986011; doi:10.1371/journal.pone.0245109)
Supplement: S2 Appendix — (DOCX) [file pone.0245109.s009.docx]

**S2 Appendix. Procedure for vegetation cover estimation within each habitat.**

- Regarding the vegetation cover of each habitat, **several variables were estimated** (Table 1):
  - quantity of light that reaches the ground.
  - Number of strata.
  - Dominant species.
  - Litter type and depth.
  - Litter humidity.
  - Soil type.
- **Dominant species of plants** within each habitat were identified at the Pontificia Universidad Javeriana Herbarium, Bogotá, D.C., (Colombia).
- **Light penetration to the ground** within each habitat was indirectly measured by the **digital camera of a cell phone (iPhone 6)**, pointing it to the sky at 1 m height. Five photos were taken and used to quantify the relative quantity of light of each one. The **estimation of the image overexposure degree** was carried out using **the Image J software**). This methodology results in an estimation of the canopy cover vegetation degree, which determines the quantity of light that penetrates to the ground and creates a shadow over it. Such quantity of light will be alter directly the relative humidity and temperature of the understory.
- The number of vegetation strata was defined by observing the mean height of **every cover vegetation type** (herbaceous, bushy or arboreal types) in five points randomly selected.
- A the **microhabitat level**, where ticks probably find a refugia, litter was observed within each habitat in order to estimate its thickness (depth to the soil) and composition according to relative fractions quantities (e.g., flowers, tree or bush branches, roots, etc.). Litter was observed in five randomly selected points. At each point, **300 to 500 g** of litter were taken, placed inside *Zip-Lock* bags and transported to the Pontificia Universidad Javeriana Herbarium. Each sample was classified according to dominant fractions and later each one was weighted (humid weight) over a digital scale. Afterward, samples were desiccated in an oven at 40ºC during three consecutive days. Then, samples were weighted again (dry weight). Weight difference represented the **quantity of water that was content within the litter**.
- At the same sampled litter sites, a small soil fraction was taken. Such sample was moistened with water and the dominant fraction (sandy, clayey or muddy soil) were estimated by manipulation with bare hands.
