# Supplementary material for: Amblyomma mixtum free-living stages: Inferences on dry and wet seasons use, preference, and niche width in an agroecosystem (Yopal, Casanare, Colombia)
Source: PLoS One. 2022 Apr 6;17(4):e0245109. doi: 10.1371/journal.pone.0245109 (PMC8986011; doi:10.1371/journal.pone.0245109)
Supplement: S3 Appendix — (DOCX) [file pone.0245109.s010.docx]

**S3 Appendix. Description of the making process for the ice traps and for the white flannelettes to collect free-living ticks on the field.**

**Making of ice traps (CO_2_):**

- A **corrugated plastic layer pad** (CartónPlast in Spanish) about 50x50 cm was used as a platform for standing one (1) ice trap.
- A **soup plate Styrofoam** was attached **double-sided tape** at the center of such a platform and **500 g of dry ice pellets** were added.
- Immediately, **a second soup plate** was placed up-side down in order **to serve as a tap**. The edges at the union of **both plates were partially attached** with small pieces of a 18 mm masking tape.
- Then, the **lower plate was thoroughly pierced** with the tip of a pencil on its lateral cylindric wall in order to facilitate **the outflow of the sublimated CO_2_** (dry ice).
- Long pieces of **double-sided tape** were thoroughly attached at each one of the four edges of the corrugated plastic layer pad (platform), on the same side were the soup plate was attached. Also, shorter pieces of double-side tape were attached at approximately 15 cm from the soup plate base framed the soup plates (see at the end of this page the S3.1Photo).

**Making of white flannelettes for dragging through transects:**

- The device for collecting tick free-living stages from grasslands was a **1.5 x 0.9 m white flannelette** attached to two aluminum tubes, 1 m long x 0.8 cm diameter each.
- This device was used in all four habitats when appropriate (see below the S3.2 Photo).

| **S3.1 Photo.** Dry ice trap placed at the ‘Riparian Forest’ habitat in summer (February/2019). | **S3.2 Photo.** White flannelette being dragged in paddock from a grassland as a test |
| --- | --- |
| **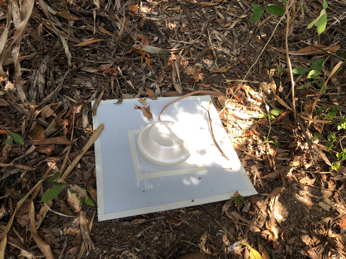** | **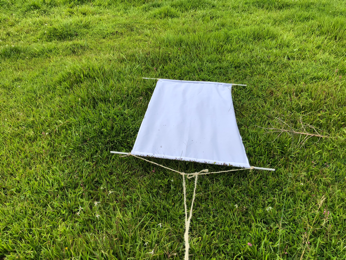** |
