# Supplementary material for: Amblyomma mixtum free-living stages: Inferences on dry and wet seasons use, preference, and niche width in an agroecosystem (Yopal, Casanare, Colombia)
Source: PLoS One. 2022 Apr 6;17(4):e0245109. doi: 10.1371/journal.pone.0245109 (PMC8986011; doi:10.1371/journal.pone.0245109)
Supplement: S4 Appendix — (DOCX) [file pone.0245109.s011.docx]

**S4 Appendix. Testing of 28 larva, nymph, and adult *Amblyomma mixtum* DNA samples (individual specimens or pools) for two mitochondrial genes and one nuclear gene.**

Initially, 206 pools of immature stage specimens collected in February/2019 were formed (35 pools of up to 30 larvae each and 97 pools of up to 16 nymphs each from CO_2_ traps, and 50 pools of up to 30 larvae each and 24 pools of up to 17 nymphs each from dragging by transects) for DNA extraction. However, PCR amplifications were unsuccessful for the next tick genes or sequences: 12S rDNA with primers TB1 (5’-AAACTAGGATTAGATACCCT-3’) and T2A (5’-AATGAGAGCGAC- GGGCGATGT-3’), COII with primers COIIF (5’-TCAGAACAYWCYTTYAATCAAAAT-3’) and COIIR2 (5’-CCACAAATTTCTGAACATTGWCCA-3’), D-Loop with primers DLIx3 (5’-TAACCGCKGCKG- CTGGCACAA-3’) and DLIx4 (5’-AGATAAYCCTTTAYTCAGGCAT-3’), and ITS-2 with primers F2LITS2 (5’-TGAGGGTCGGATCAYATATCA-3’) and McLn (5’-GTGAATTCTATGCTTAAATTC- AGGGGGT-3’). Because of that new DNA extractions were carried out on 21 samples representing all tick stages from the collected specimens and four samples (one female, one male, one nymph, and one larvae) were coupled to cPCR assays and sequencing for three genes (16S rRNA, COI, and ITS-2). However, from 17 samples up to seven new additional sequences from immature tick stages (up to five of larvae and two of nymphs) were obtained for the ITS-2 and the COI genes from the initial 206 pools of extracted DNA. No additional 16S rRNA gene sequences were obtained as the previous ones grouped all the three tick stages for this gene. In total, eleven amplicons were coupled to sequencing. The methodology and subsequent results are described in the main document. The agarose gel electrophoresis assays for each gene are shown in photos S4.1 to S4.6.

| **S4.1 Photo.** Agarose gel electrophoresis showing the cPCR amplification of the 16S rRNA gene from 15 *A. mixtum* adult stage DNA samples. | **S4.2 Photo.** Agarose gel electrophoresis showing the cPCR amplification of the 16S rRNA gene from *A. mixtum* nymph and larva DNA pools. |
| --- | --- |
|  |  |
| MP = molecular marker; bp = base pairs; NC = negative control (molecular grade sterile water); no positive control was included in this gel because it was intended only for showing purposes. 15 DNA randomly selected samples from females and males individual or pool specimens are shown. | MP = molecular marker; bp = base pairs; neither negative nor positive controls were included as this gel was intended only for showing purposes; N1 = nymph DNA pool 1; N2 = nymph DNA pool 2; N3 = nymph DNA pool 3; L1 = larva DNA pool 1; L2 = larva DNA pool 2; and L3 = larva DNA pool 3. Each nymph pool had six specimens, while L1 had 30 larva, L2 had 31 larvae, and L3 had 34 larvae. |

| **S4.3 Photo.** Agarose gel electrophoresis showing the qPCR amplification of the COI gene from 11 *A. mixtum* adult and immature stage DNA samples. | **S4.4 Photo.** Agarose gel electrophoresis showing the cPCR amplification of the COI gene for the 10 remained *A. mixtum* adult stage DNA samples. |
| --- | --- |
|  |  |
| MP = molecular marker; bp = base pairs; CN-R = PCR assay negative control (molecular grade sterile water); CN-E = extraction negative control (free recipient with only DNA extraction reagents); CP = positive control (*Amblyomma patinoi* DNA adult stage sample). L1-L3 = larva DNA pools; N1-N3 = nymph DNA pools; V7-V95 = Adult stages (female and males) DNA individual specimens or pool samples. | MP = molecular marker; bp = base pairs; neither negative nor positive controls were included as this gel was intended only for showing purposes; V4-V108 = Adult stages (female and males) DNA individual specimens or pool samples. |

**S4.5 Photo.** Agarose gel horizontal electrophoresis showing the cPCR amplification of the ITS-2 gene from 21 selected DNA samples.

MP = molecular marker; CN-R = PCR assay negative control (molecular grade sterile water); CN-E = extraction negative control (free recipient with only DNA extraction reaagents); CP = positive control (*Amblyomma patinoi* DNA adult stage sample); L1-L3 = larva DNA pools; N1-N3 = nymph DNA pools; V7-V100 = Adult stages (female and males) DNA individual samples.

**S4.6 Photo.** Agarose gel horizontal electrophoresis showing the cPCR amplification of the ITS-2 the COI-1 genes from seven additional selected DNA samples.


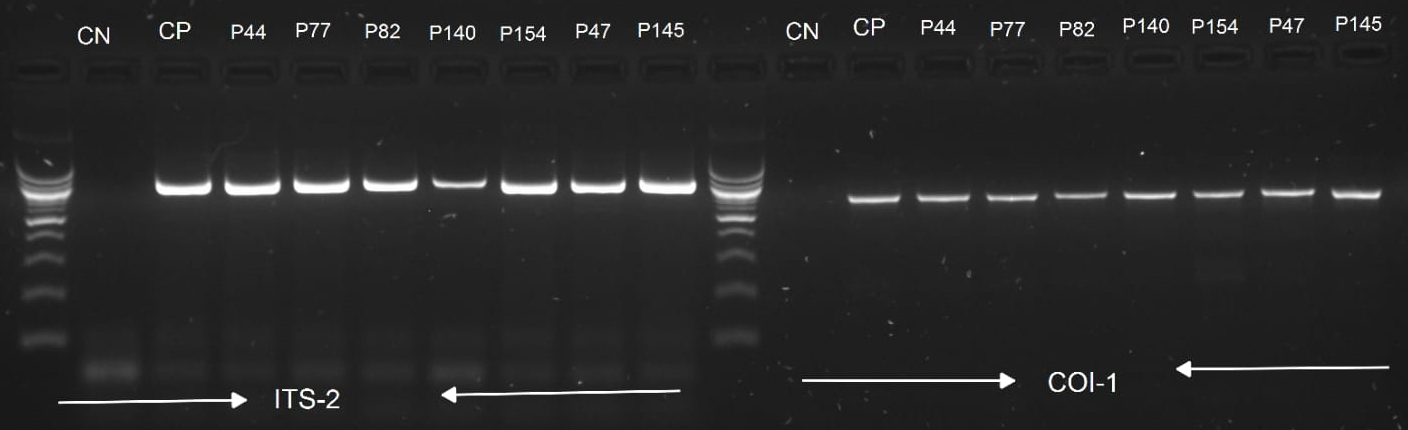


The first unlabeled column corresponds to the molecular marker, which is identical to S3-S5 images. CN = PCR assay negative control (molecular grade sterile water); CP = positive control (*Amblyomma patinoi* DNA adult stage sample); P44, P77, P82, P140, and P154 = larva DNA pools; P47 and P145 = nymph DNA pools.
